# Supplementary material for: A chromosome-level genome assembly of Cydia pomonella provides insights into chemical ecology and insecticide resistance
Source: Nat Commun. 2019 Sep 17;10:4237. doi: 10.1038/s41467-019-12175-9 (PMC6748993; doi:10.1038/s41467-019-12175-9)
Supplement: Supplementary file 1 — Supplementary Information [file 41467_2019_12175_MOESM1_ESM.pdf]

Supplementary information for:

A chromosome-level genome assembly of *Cydia pomonella* provides insights into chemical ecology and insecticide resistance

Fanghao Wan[1,2 ,#,*], Chuanlin Yin[3,#], Rui Tang[4,5,#], Maohua Chen[6,#], Qiang Wu[1,#], Cong Huang[1,7,#], Wanqiang Qian[2], Omar Rota-Stabelli[8] ,Nianwan Yang[1,*], Shuping Wang[9], Guirong Wang[1], Guifen Zhang[1], Jianyang Guo[1], Liuqi (Aloy) Gu[10], Longfei Chen[3], Longsheng Xing[2], Yu Xi[2], Feiling Liu[3], Kejian Lin[1], Mengbo Guo[1], Wei Liu[1], Kang He[3], Ruizheng Tian[6], Emmanuelle Jacquin-Joly[12], Pierre Franck[13], Myriam Siegwart[13], Lino Ometto[8,22], Gianfranco Anfora[8,14], Mark Blaxter[15], Camille Meslin[12], Petr Nguyen[16,17], Martina Dalíková[16,17], František Marec[16], Jérôme Olivares[13], Sandrine Maugin[13], Jianru Shen[1], Jinding Liu[11], Jinmeng Guo[11], Jiapeng Luo[3], Bo Liu[2], Wei Fan[2], Likai Feng[18], Xianxin Zhao[3], Xiong Peng[6], Kang Wang[6], Lang Liu[6], Haixia Zhan[4], Wanxue Liu[1], Guoliang Shi[1,19], Chunyan Jiang[1,19], Jisu Jin[1,7], Xiaoqing Xian[1], Sha Lu[1,19], Mingli Ye[20], Meizhen Li[3], Minglu Yang[21], Renci Xiong[21], James R Walters[10,*], Fei Li[3,*]

Contents

Supplementary Methods 6

1. Genome sequencing 6

1.1 Insects.. 6

1.2 Flow Cytometry, genome size and heterozygosity estimation.. 6

1.3 Illumina HiSeq and PacBio sequencing.. 6

1.4 BioNano and Hi-C sequencing 7

1.5 Nanopore sequencing and analysis 8

1.6 Full-length transcripts sequencing and analysis.. 8

1.7 Transcriptome sequencing.. 9

2. Genome assembly 9

2.1 Draft genome assembly.. 9

2.2 BioNano 10

2.3 Hi-C-based proximity-guided assembly 10

2.4 Genome assessment 11

3. Genome annotation 12

3.1 Identifying repeat sequences.. 12

3.2 Annotating protein coding genes with OMIGA.. 13

3.3 Gene function assignment.. 13

3.4 Noncoding RNA gene annotation 14

3.5 Ortholog predictions 14

3.6 Phylogenetic analysis.. 15

4. Synteny, karyotype, and sex chromosomes 16

4.1 Whole Genome alignments.. 16

4.2 Detection of sex chromosomes 16

4.3 Repeat Analysis.. 17

4.4 Analysis of sex chromosomes.. 17

5. Odorant receptors in *C. pomonella* 20
5.1 Identify odorant receptors of *C. pomonella* 20
5.2 Phylogenetic analysis..........20
5.3 Sequence alignment and gene structure of *CpomOR3* and *CpomOR3a* genes .21
5.4 Gene expression analysis 21
5.5 *In situ* hybridization 21
6. Functional analysis of *CpomOR3a* and *CpomOR3b*..........22
6.1 Insects..........22
6.2 Chemicals..........22
6.3 RNA extraction and cDNA synthesis..........23
7. Genome re-sequencing..........24
7.1 Insects for genome re-sequencing..........24
7.2 Re-sequencing procedure..........25
7.3 GWAS analysis of different strains which susceptible or resistance to insecticides 25
8. SNPs validation and RNA interference..........26
8.1 Insects & Chemicals..........26
8.2 SNPs validation..........27
8.3 siRNA injection..........27
8.4 Quantitative PCR 28
8.7 Insecticide bioassay..........29
Supplementary Figures 30
Supplementary Figure 1 30
Supplementary Figure 2 32
Supplementary Figure 3 33
Supplementary Figure 4 34
Supplementary Figure 5 35

Supplementary Figure 6 36
Supplementary Figure 7 38
Supplementary Figure 8 39
Supplementary Figure 9 40
Supplementary Figure 10 41
Supplementary Figure 11 42
Supplementary Figure 12 43
Supplementary Figure 13 44
Supplementary Figure 14 45
Supplementary Figure 15 46
Supplementary Figure 16 47
Supplementary Tables 48
Supplementary Table 1 48
Supplementary Table 2 54
Supplementary Table 3 55
Supplementary Table 4 56
Supplementary Table 5 58
Supplementary Table 6 59
Supplementary Table 7 60
Supplementary Table 8 61
Supplementary Table 9 62
Supplementary Table 10 63
Supplementary Table 11 64
Supplementary Table 12 65
Supplementary Table 13 66
Supplementary Table 14 67
Supplementary Table 15 68

Supplementary Table 16..69
Supplementary Table 17..70
Supplementary Table 18..71
Supplementary Table 19..72
Supplementary Table 20..73
Supplementary Table 21..74
Supplementary Table 22..75
Supplementary Table 23..76
Supplementary Table 24..77
Supplementary Table 25..78
Supplementary Table 26..79
Supplementary Table 27..80
Supplementary References...81

Supplementary Methods

1. Genome sequencing

1.1 Insects

The *C. pomonella* were collected at Jiuquan city, Gansu province in December 2013 (Jiuquan strain), and then maintained by an artificial diet in the laboratory of the Chinese Academy of Inspection and Quarantine. The insectarium environment was set at 25±1°C and 75±5% relative humidity on a photoperiod (Light: Dark = 14:10).

1.2 Flow Cytometry, genome size and heterozygosity estimation

Flow cytometry as well as K-mer based analysis of the whole genome shotgun sequencing were used to estimate the genome size and heterozygosity of the *C. pomonella*. In the flow cytometry experiment, we selected the *Drosophila melanogaster* Canton-S strain adults (176.4 Mb) as the reference species (**Supplementary Fig. 2**). The thoracic tissue of adult females of *C. pomonella* was used to measure the genome size. The flow cytometry experiments were performed following the procedures described previously[1].

For K-mer analysis, genomic DNA was extracted from three female fifth instar larvae which were maintained by sibling mating for four generations. Two Illumina PE libraries (180 and 500 bp) were constructed and sequenced with Illumina HiSeq 2000 platform. In total, 137 Gb HiSeq clean data were obtained. The distribution of K-mers depends on the characteristic of the genome and follow a Poisson's distribution[2]. A K-mer refers to an artificial sequence division of K nucleotides iteratively from sequencing reads. To obtain independent estimates of genome size and repeat content we used the software JELLYFISH (version 2.2.6)[3] to generate k-mer spectra of original the raw sequencing data with the default parameters (**Supplementary Tables 2 and 3; Supplementary Fig. 3**).

1.3 Illumina HiSeq and PacBio sequencing

For Illumina HiSeq sequencing, genomic DNA was extracted from 42 fifth instar female larvae of an inbred Jiuquan strain which was maintained by sibling mating for six generations. To decrease the risk of non-randomness, we built different insert sizes libraries. Four paired-end sequencing libraries of *C. pomonella* (180 bp, 300 bp, 500 bp and 800 bp) and 3 mate-pair sequencing libraries (3 Kb, 8 Kb and 10 Kb) were constructed, respectively. All libraries were sequenced by using Illumina HiSeq 2000 101PE platform. In total, we obtained ~300 Gb raw data. After filtering out low quality and duplicated reads, 245.5 Gb clean data were maintained for genome assembly (**Supplementary Table 2**).

For PacBio sequencing, genomic DNA was extracted from 22 individuals of fifth instar female larvae. We generated 54.57 Gb data sequenced for 38 cells by the Pacbio RS II sequencing platform at the Annoroad Gene Technology Co. Ltd (**Supplementary Table 4**).

1.4 BioNano and Hi-C sequencing

To obtain a high-quality genome assembly, the BioNano next-generation mapping system was used. A total of 3,000 newly hatched larvae were collected. Scaffolding of the contigs/scaffolds with optical mapping was performed using the Irys optical mapping technology (BioNano Genomics). Purified DNA was embedded in a thin agarose layer and was labeled and counterstained following the IrysPrep Reagent Kit protocol (BioNano Genomics). Samples were then loaded into IrysChips and run on the Irys imaging instrument (BioNano Genomics). Single molecules under 150kb in size or with fewer than 500 labels were removed. An optical map of the sample was produced in two instrument runs with labeled single molecules. These experiments were carried out at the Annoroad Gene Technology Co. Ltd.

Next, we used Hi-C data to detect the chromosome contact information for assisting genome assembly. The crosslinking of samples was performed as follows: ~500 second instar larvae were cut with scissors to produce incisions, after which 1.25 ml of 37% formaldehyde were added to obtain 2% final concentration for crosslink. The samples were

mixed gently immediately after addition of formaldehyde, incubated at room temperature (RT) for 10 min on plates that were gently rotated every 2 min. Then, 2.5 ml of 2.5 M glycine was added to quench the crosslink, mixed well and incubated at RT for 5 min, and then incubated on ice for 15 min to stop crosslinking completely. Finally, samples were centrifuged at 2,000 g for 10 min at 4 °C, and the supernatant was removed with a pipette. After crosslinking, the samples were used for quality control, Hi-C library preparation and sequencing using Illumina HiSeq platform with 2×150-bp reads (**Supplementary Table 6**). All experiments and analysis were carried out at the Annoroad Gene Technology Co. Ltd (**Supplementary Fig. 15**).

1.5 Nanopore sequencing and analysis

For the Nanopore sequencing, genomic DNA was extracted from 10 female pupa using the QIAamp DNA mini kit (Qiagen), and one 20 Kb insert size library was prepared according to the SQK-LSK108 1D ligation genomic DNA protocol. After library preparation, the DNA was transformed to a flow cell and sequenced in the PromethION Oxford Nanopore Technology sequenator by Nextomics Biosciences company. If the sequencing reads contained the adaptor or mean sequencing quality score less than 7 will be removed. The minimap2 software was used to align the long reads to the reference genome.

1.6 Full-length transcripts sequencing and analysis

The larva from 2-instar to 5-instar, pupa and adult of codling moth were collected and mixed, then the total RNA was extracted by TRIzol. Subsequently, poly(A) RNA enrichened by Oligo(dT) was reverse transcribed into cDNA using the SMARTer® PCR cDNA Synthesis Kit. The library preparing and sequencing with one SMRT cells on the PacBio RSII platform were carried out at the Annoroad Gene Technology Co. Ltd according to the standard manufacturer's instructions. After sequencing, the polymerase reads with adaptor contaminated or which length less than 50 bp and the precious score

smaller than 0.8 will be removed. Then the polymerase reads data were analyzed by the RS_IsoSeq analysis pipeline (**https://github.com/ben-lerch/IsoSeq-3.0**). The FLNC CCSs were mapped against to the codling moth genome by the GMAP software and the MachAnnot software were adopted to compare the pacbio full-length transcripts with the genome gene structure annotation.

1.7 Transcriptome sequencing

The eggs of codling moth were collected at one day and four days after laying. For the larva, we collected the 5th-instar larva, mature larva of female and male. For the pupae, only females were collected. We also collected heat-treated female adult individuals, and abdomens from female and male adults raised under standard conditions. In total, ten samples were collected from the laboratory population of codling moth and sent to the Shenzhen Millennium Spirit Technology Co. Ltd., China for transcriptome sequencing. All the libraries of the samples were prepared followed by TruSeq RNA Sample Preparation v2 Guide, Part #15026495 Rev.F protocol, and the reagent are TruSeq rapid SBS kit or Truseq SBS Kit v4. All the samples were sequenced at the Illumina Hiseq 2500 with the read length 101, and the BCL (base calls) binary is converted into FASTQ utilizing illumina package bcl2fastq (v1.8.4). After removing low quality or contaminating reads, a total of 54 Gb clean data were obtained (**Supplementary Table 10**).

2. Genome assembly

2.1 Draft genome assembly

The draft genome was assembled using the raw reads of the PacBio and Illumina sequencing platform. First, we filtered low-quality reads. For the Illumina reads, the filtering criteria were: 1) adaptor contamination reads (the adaptor contamination reads length >5bp); 2) Low sequencing score reads (The percentage of bases which the Q value is less than 19 >=15%); 3) N enrichment reads (The percentage of N bases >5%). For the

paired-end reads, if one-side is identified to be a low-quality read, both paired reads will be removed. For the PacBio reads, the filter criteria are: 1) The shorter length reads; 2) Low sequencing score reads; 3) adaptor contamination reads.

We used different methods in combining PacBio and Illumina data to assemble the draft genome. We compared the results of different methods, and finally chose the method using PacBio to assemble the frame of the draft genome scaffolds and then polish and improve the scaffolds with Illumina clean reads. To assemble the draft genome scaffolds from the PacBio reads, we used the Falcon v0.3.0 software[4]. We used the Redundans[5] software to remove redundant scaffolds from the assembly and generate a non-redundant assembled genome. Finally, the illumina data were used to correct the genome assembly by the Pilon software (https://github.com/broadinstitute/pilon)[6].

2.2 BioNano

The IrysView (BioNano Genomics) software package was used to produce single-molecule maps and *de novo* assemble maps into a genome map with default parameters. Hybrid Scaffolds were assembled by hybrid Scaffold pipeline from Bionano Solve software package with default parameters.

2.3 Hi-C-based proximity-guided assembly

The processed information by Illumina high-throughput was restored as raw image data format and would be recognized and transformed to sequenced reads. These reads could contain some adapters, low quality calling bases. To avoid alignment error, raw reads were filtered and trimmed, and only reads passing this cleaning stage were used in subsequent analyses. The filtering criteria are: (1) Trim adapter contamination from reads (use adapter sequence information to trim reads); (2) Remove the low-quality reads (remove reads with base calling quality $Q \leq 19$); (3) Remove reads with N percentage >5% (For pair-end sequencing, if one end has N% >5%, both ends will be removed).

Cleaned reads were first aligned to the reference genome using the bowtie2 end-to-end algorithm[7]. Unmapped reads are mainly composed of chimeric fragments spanning the ligation junction. According to the Hi-C protocol and the fill-in strategy, Hi-C-Pro (V2.7.8)[8] was used to detect the ligation site using an exact matching procedure and to align back on the genome the 5′ fraction of the read. The results of two mapping steps are then merged in a single alignment file. Low mapping quality reads, multiple hits and singletons were discarded.

We removed duplicated reads and kept reads that uniquely mapped to the reference genome. The assembly package, Lachesis, was applied to do clustering, ordering and orienting. Based on the agglomerative hierarchical clustering algorithm[9], we clustered the scaffolds into N groups. Then, the longest acyclic spanning tree, called "trunk", was built according to the relations between the normalized Hi-C interactions and the scaffolds that were excluded from the trunk were reinserted into it at sites that maximized the amount of linkage between adjacent scaffolds. For each chromosome cluster, we got an exact scaffold order of the internal groups and traversed all the directions of the scaffolds through a weighted directed acyclic graph (WDAG) to predict orientation for each of the scaffolds. The contacts of intra-chromosome are stronger than that of the inter-chromosome, and the interactions decrease with the distance in a chromosome. Corresponding to these two rules, the interactions near the diagonal line are obviously stronger than those locating apart from the diagonal line and close bins have a strong relationship in a heatmap. We cut the chromosomes which predicted by Lachesis into bins with equal length such as 1Mb or 500Kb and constructed heatmap based on the interaction signals that revealed by valid mapped read pairs between bins. If the heatmap didn't conform to these rules, it suggested there must be something wrong in the assembly result (**Supplementary Fig. 15**).

2.4 Genome assessment

CEGMA (version 2.4)[10] and BUSCO (version 3.0)[11] were used to estimate the

completeness of the codling moth genome assembly. To run BUSCO software, we selected the insecta db 9 datasets (http://busco.ezlab.org/v2/datasets/insecta_odb9.tar.gz) as the library which contains 1658 benchmarking universal single-copy orthologous genes. Both CEGMA and BUSCO were performed with default parameters. To compare the gene space of the codling moth genome with other species, we collected all the published insect genomes and used the same parameters and procedures to assess them. The results proved that the genome assembly of the codling moth had a high quality (**Table 1; Supplementary Table 8**).

To further validate the reliability and completeness of the genome assembly, we sequenced genomic DNA with Oxford Nanopore platform, yielding ~71 Gb data. Aligning the Nanopore reads to the reference genome showed that 99.96% reads can be mapped with the genome scaffolds. There are 6,070 reads whose lengths are larger than or equal to 100 Kb, and these ultra-long reads can be uniquely mapped to the genome scaffolds with high consistency. In addition, we sequenced full-length transcripts with PacBio platform and obtained totally 37.57 Gb with 704,348 polymerase reads, yielding 500,583 full-length non-chimeric (FLNC) circular consensus sequence (CCS) subreads with the mean length of 2343,32 bps. We finally got 25,940 high quality consensus isoform transcripts and 15,260 protein coding transcripts with complete open read frame (ORF), with the mean lengths of 2,571.98 and 1,239.45 bp, respectively. More than 93% full-length transcripts could be exclusively mapped to the reference genome.

3. Genome annotation

3.1 Identifying repeat sequences

To reduce the complication in genome annotation[12], repeat sequences were masked. Tandem Repeats Finder (TRF) was used to search tandem repeats in the genomes[13], and novel repeat sequences were predicted by RepeatModeler (version 1.0.7)[14], which includes two *de novo* programs, RECON (version 1.08)[15] and RepeatScout (version 1.0.5)[16].

Transposable elements (TEs) were predicted in the assemblies by homology searching against RepBase using RepeatMasker (version 4.0.5)[17]. Both programs were used with default parameters.

3.2 Annotating protein coding genes with OMIGA

We used OMIGA[18] to annotate the codling moth genome by integrating evidence from homolog searching, transcriptome sequencing, and *de novo* predictions. Sequences of homologous proteins were downloaded from the NCBI invertebrate RefSeq. The transcriptome assembly were used to provide gene expression evidence which was assembled followed the protocol described by Trapnell[19]. Three *ab initio* gene prediction programs including Augustus (version 3.1)[20], SNAP (version 2006-07-28)[21] and GeneMark-ET (Suite 4.21)[22] were used for *de novo* gene prediction. To obtain high accuracy, *de novo* gene prediction software must be re-trained. We selected the transcripts with intact open reading frame (ORF) from the transcriptome to re-train Augustus and SNAP classifiers. To determine the transcripts with intact ORF, we used the BLAST search against the UniProtKB/Swiss-Prot proteins database (E-value =1e-5) and Pfam to identify protein domains. After filtered by TransDecoder (http://transdecoder.sourceforge.net/) software, only the transcripts with a complete ORF were included. If genes had multiple transcripts, only the longest transcript was remained. Then, these gene transcripts were used to re-train the prediction software Augustus and SNAP. For GeneMark-ET, the whole assembly which more than 10 Mb were used to re-train the software. All gene evidence identified from above three approaches were combined by MAKER pipeline (version 2.31)[23] into a weighted and non-redundant consensus of gene structures. The default parameters were used for MAKER.

3.3 Gene function assignment

To assign functions to annotated protein-coding genes, we used these genes as queries to

BLASTP against UniProtKB/Swiss-Prot proteins or NCBI Non-redundant protein sequences (nr). The E-value cutoff was set as 1e-5. The best 20 hits were used for function assignment. Protein domains were annotated by InterProScan (version 5.21-60.0)[24] with the pather data version 10.0. A Gene Ontology (GO) term for each gene were obtained by the software Blast2GO[25], and simplification of the annotation into functional categories was also done by Blast2GO using GO slim. Proteins were summarized at level two into three main GO categories (biological process, cellular component, and molecular function). The KEGG pathway annotation were carried out by the BlastKOALA web server (https://www.kegg.jp/blastkoala/), and also Clusters of Orthologous Groups of proteins (COGs) were annotated by in-house Perl scripts.

3.4 Noncoding RNA gene annotation

Three types of ncRNAs, transfer RNA (tRNA), ribosomal RNA (rRNA), and small nuclear RNA, were annotated. To identify ncRNAs, the sequences of protein coding genes, repetitive elements and other classes of non-coding RNAs were removed from the genome Scaffolds. tRNA genes were predicted by tRNAscan-SE[26] with eukaryote parameters. rRNA fragments were identified by aligning the rRNA template sequences from invertebrate animals to genomes using BLASTN with an E-value cutoff of 1E-5. Small nuclear RNA genes were inferred by the INFERNAL software against Rfam database of release 11.0[27]. The MapMi program (version 1.5.0)[28] was used to identify the miRNA homologs by mapping all miRNA matures in the miRBase[29] against the codling moth genome, and mirdeep2 software was used to identify novel miRNAs in the small RNA data. All algorithms were performed with default parameters.

3.5 Ortholog predictions

Orthologous groups were constructed with OrthoMCL pipeline[30] using the protein sequences of *C. pomonella*, another six Lepidoptera insects (*Danaus plexippus, Heliconius*

melpomene, Melitaea cinxia, Manduca sexta, Bombyx mori, Trichoplusia ni, Spodoptera litura, Plutella xylostella), two Diptera species (*D. melanogaster* and *A. gambiae*), two Hemiptera species (*Rhodnius prolixus, Bemisia tabaci*), two Hymenoptera insects (*N. vitripennis* and *A. mellifera*), two Coleoptera species (*T. castaneum* and *Anoplophora glabripennis*), two Isoptera species (*Cryptotermes secundus, Zootermopsis nevadensis*), as well as one Orthoptera species (*Locusta migratoria*) (**Supplementary Table 16**). The default parameters were used in the pipeline, and then the orthologous groups were assigned by an in-house python script.

3.6 Phylogenetic analysis

We constructed a phylogenetic tree of *C. pomonella* and other 19 selected insects (*D. plexippus, H. melpomene, M. cinxia, M. sexta, B. mori, T. ni, S. litura, P. xylostella, D. melanogaster, A. gambiae, T. castaneum, A. glaringness, N. vitripennis, A. mellifera, R. prolixus, B. tabaci, C. secundus, Z. nevadensis, L. migratora*) using 1:1 single-copy orthologous genes. Phylogeny was inferred on the concatenated 500 orthologs dataset (including 452467 amino acids, aa) after excluding poorly conserved sites using Gblocks with default parameters (59621 final aa dataset). We used RaXml employing an LG+G replacement model and bootstrapped the dataset using 100 pseudo-replicates. We inferred divergence times using the Bayesian method implemented in Phylobayes using the Raxml tree topology, an LG+G replacement model, a Birth Death tree prior, and a relaxed lognormal clock. The relaxed clock was preferred over a strict clock by comparison of the harmonic mean of the likelihood (and AICM) ona preliminary analysis in BEAST 2. We calibrated sequences using two fossil calibration previously described[31]: The *Drosophila - Anopheles* split with a minimum at 238.5 Mya, and a soft maximum at 295.4 Mya, and the *Spodoptera – Bombyx* split with a minimum at 48.4 Mya and a maximum at 200.2 which is regarded as Early Eocene (Ypresian: 55.8 +/-0.2 to 48.6+/-0.2), and from the oldest lepidopteran Archaeolepis, from the Early Jurassic of Dorset which is Hettangian to

Sinemurian, with an age range from 196.5 +/- 1.0 to 189.6 +/-1.5 and the age range for the Hettangian of 199.6 +/-0.6 to 196.5 +/- 1.0[31]. We set flat probabilities between these two boundaries allowing soft bounds with a soft tail of 5% to both boundaries. We used as root prior the posterior estimate for the split of Ortohopteroidea from Holometabola at 385 Mya with standard deviation 10[32]. We set two independent analyses and harvest trees after a burn-in of 20% and after assessing that the two analyses converged after 20.000 generations on very similar estimates.

4. Synteny, karyotype, and sex chromosomes

4.1 Whole Genome alignments

Whole genome alignments were generated using Satsuma with default values[33].

4.2 Detection of sex chromosomes

We compared sequencing coverage differences between male and female samples in order to detect sex-linked regions of the genome. Cytogenetic analysis reports substantial differentiation of the Z and W chromosome, thus we expect distinct patterns of Illumina sequencing coverage between sexes on the Z, W, and autosomes. Specifically, autosomes should have equal coverage while the Z should show an approximately two-fold greater coverage in males. The W should show a strongly female-biased coverage pattern, but the precise ratio is difficult to estimate because the W chromosome may contain regions of substantial sequence similarity to autosomes or the Z due either to shared repetitive sequences or homology to the neo-Z.

The samples from the S population, providing three individuals of each sex, were aligned to the reference genome with bowtie. Read counts were tallied per scaffold, normalized by median sample coverage, and averaged by sex to give a single representative coverage value per scaffold for each sex. Additionally, scaffolds were similarly analyzed using non-overlapping 500 bp windows in which to count and average reads and calculate

male:female coverage.

4.3 Repeat Analysis

Repetitive regions of the genome assembly corresponding to the de novo library from RepeatModeler[14] as well as Arthropod repeats in RepBase[34] were identified, classified, and quantified using RepeatMasker[35].

Female-enriched repeats were assessed using the RepeatExplorer[36, 37] pipeline. Illumina PE reads from three females and three males were quality filtered (plus adaptors were removed) and trimmed to uniform length of 130bp. Random samples of PE reads from all six individuals (total used coverage was 0.2-1.2x) were applied to RepeatExplorer pipeline. To assess the presence of individual repeats in females and males average, read count from three individuals was considered for each cluster formed by RepeatExplorer. If the mean read counts differed significantly between sexes via t-test, the log2 of female/male mean read counts were calculated and plotted. The entire analysis was performed seven times, (employing seven different random samplings) and only repeats female-enriched in more than four analyses were considered. These repeats were annotated based on graph topology (globular graphs are typical for tandem repeats), Repeat Explorer predictions, and homology search (blastx, RepeatMasker).

4.4 Analysis of sex chromosomes

We confirmed the presence in our assembly of the Z chromosome, and a large portion of the W, through sex-specific patterns of sequencing coverage (**Fig. 2**, main text). All but two chromosomal-length scaffolds showed equal coverage between sexes, as expected for autosomes. The largest scaffold (chr1) yielded two-fold greater male coverage, as expected for the Z chromosome. This two-fold difference is consistent across both the ancestral and neo portions of the Z (**Fig. 2**, main text), indicating very little remaining sequence homology, if any, between the neo-Z segment and the current W sequence, as suggested by prior cytogenetic work[38]. If substantial homology persisted such that W-linked sequences would

map to the neo-Z at any appreciable rate, then this ratios in this segment of the Z should be notably shifted towards one for such regions, a pattern that is not observed here.

In contrast, the chr29 scaffold showed a strongly female-biased coverage ratio, indicating it represents W-linked sequence. The pattern of male:female coverage is much more variable across the chr29 scaffold than for other chromosomes (**Fig. 2**). This likely reflects the abundance of TEs on the W which are variably collecting read mappings from TEs in other regions of the genome. Prior cytogenetic analyses indicate the W chromosome is approximately the same size as the Z[38, 39], while the chr29 scaffold is only about 1/10 the size the chr1 scaffold, suggesting that this scaffold represents only a small portion of the entire W chromosome. Several smaller scaffolds not yet assigned to chromosome have strongly female-biased coverage values and may also reflect additional W chromosomal content (**Fig. 2**), but coverage-ratio point estimates for short scaffolds are highly variable and do not provide high confidence assessment of sex-linked regions. Yet even including these smaller, female-biased (>2x F:M coverage) scaffolds only adds ~1 Mbp to the total putative W-linked sequence represented in the assembly. Nonetheless, the chr29 scaffold alone still provides >5 Mbp of contiguous W-linked sequence, which is more than has been reported in any species of Lepidoptera.

The Z:autosome fusion in *C. pomonella* raises questions concerning the fate of the maternally inherited autosomal homolog following the fusion event. Cytogenetic analysis revealed no evidence of shared sequence between the Z and W, suggesting nearly complete degeneration of homologous W alleles[39]. We sought to complement this cytogenetic analysis with bioinformatic homology searches, which were conducted at a variety of scales. First we performed nucleotide and translated amino-acid alignments between chr1 and chr29 scaffold with MUMmer using the NUCmer and PROmer algorithms, respectively[40]. We also performed nucleotide alignments of these scaffolds using Satsuma[33]. All approaches yielded only very short, scattered, and repeated segments of similarity as would be expected from homology due transposable elements (**Supplementary Fig. 8**). There was no obvious pattern

of global collinearity or homology between chr1 and chr29 revealed by these attempts at alignment.

Additionally, we specifically sought to detect gametologs between the Z and W by tBLASTn searches of W-linked protein sequences against the chr1 scaffold. About 500 protein-coding genes were predicted on chr29 by automated annotation. However, comparison of these proteins to sequences in RepBase[34] and further functional annotation strongly suggested the vast majority of these predicted proteins are components of transposable elements and not of significant organismal function. Only 27 predicted chr29 proteins appeared to lack obvious indications of association with transposable elements. However, BLAST searches across the remainder of chromosomal scaffolds returned several strong hits distributed across the genome for each these proteins, indicating that these proteins also likely correspond to transposable elements; there was no evidence for these having unique Z-linked gametologs. Thus, primarily through the absence of any strong detectable homology between the Z and W sequences in the *C. pomonella* assembly, we confirm the substantial degradation or loss of the W chromosome in the *C. pomonella* lineage.

We further explored various sequence characteristics of chr29 relative to the rest of the genome. The proportion of GC is slightly elevated compared to than other chromosomes (**Supplementary Fig. 9**). Lepidopteran W chromosomes are typically highly degenerate, being gene-poor while repeat-rich. Chr29 does indeed appear to be gene-poor; as mentioned above, we detected no chr29 protein-coding genes that appear to be anything other than TEs. However, results from repeat masking do not indicate notably greater repeat content than other chromosomes, though the structure and composition of W-linked repeats do appear distinct (**Supplementary Figs. 10 and 11**). W repeats are considerably fewer in total number but are longer in average length compared to the other chromosomes. Also, the W hosts a notably larger proportion of long terminal repeat (LTR) and DNA transposons compared to the other chromosomes (**Supplementary Fig. 11**). Analysis of sex-specific Illumina data via RepeatExplorer[37] identified four repeats that were significantly enriched in females

(**Supplementary Fig. 12; Supplementary Table 17**).

5. Odorant receptors in *C. pomonella*

5.1 Identify odorant receptors of *C. pomonella*

We collected protein sequences of previously reported *Or* genes from several species of Lepidoptera with published genome data: *Bombyx mori*[41-45], *Manduca sexta* [46], *Plutella xylostella*[45] and *Danaus plexippus*[47]. These protein sequences were used as queries to perform BLASTP search (e-value cutoff of 1e-5) against the *C. pomonella* genome to find the candidate *Or* genes. A local command line HMMER (version 3.1b2) search[48] for these candidate *Or* genes used Pfam-A database[49] to find 7tm_6 (PF02949) or 7tm_4 (PF13853) HMM profile. The sequences contain the HMM profile was regarded as the certain *Or* genes. The transmembrane helix was analyzed using TMHMM (version 2.0)[50]. Then, we used an in-house bioinformatics pipeline previously described[51] to find new candidate *Or* genes in *C. pomonella*.

5.2 Phylogenetic analysis

To reconstruct the phylogenetic tree of the whole *Or* gene family, we first aligned all 311 reference protein sequences using MAFFT software[52] with the default option, the alignment was trimmed using trimAl v1.4[53] to remove low-quality regions based on a heuristic approach (-automated1) that depends on a distribution of residue similarities inferred from the alignment for *Or* gene family, and then, a maximum-likelihood tree was performed using RAxML (v8.1.16)[54] with an amino acid substitution model "PROTGAMMAJTTF" inferred from Prottest3 and 1000 bootstrap replicate searches. Finally, the trees were prepared in iTOL v4.2 (http://itol.embl.de/) and Adobe Illustrator (Adobe Systems, San Jose, CA, USA). Similar protocol was followed for establishment of phylogenetic trees for other gene families including *OBPs*, *CSPs*, *GRs*, *IRs*, and *SMNPs* in *C. pomonella*.

Phylogenetic tree of *Ors* was established using genes from *Cydia pomonella*, *Bombyx mori*, *Danaus plexippus*, *Helicoverpa armigera*, and *Manduca sexta*. Phylogenetic trees of other gene families were established using genes from *Cydia pomonella* (Cpom), *Bombyx mori* (Bmor), *Danaus plexippus* (Dple), *Helicoverpa armigera* (Harm), *Manduca sexta* (Msex), *Drosophila melanogaster* (Dmel), *Amyelois transitella* (Atra), *Plutella xylostella* (Pxyl), *Spodoptera litura* (Slit), and *Trichoplusia ni* (Trni)

5.3 Sequence alignment and gene structure of *CpomOR3* and *CpomOR3a* genes

The protein sequences of *CpomOR3a* and *CpomOR3b* were aligned in GeneDoc to compare sequence similarity (**Supplementary Fig. 13**). We drew the gene structure of these two genes using an online website Exon-Intron Graphic Maker (**http://wormweb.org/exonintron)**, respectively. And the relative position in chromosome was drawn in Adobe Illustrator.

5.4 Gene expression analysis

The antennal transcriptome data of *C. pomonella* were obtained from NCBI: Adult male antennae (SRX1082029), adult female antennae (SRX1082030) and neonate larval heads (SRX1082032). Gene expression levels were calculated by RSEM software[55] using the fragments per kilobase of exon model per million mapped fragments (FPKM) method based on the results of antennal transcriptome analysis. The number of fragments that uniquely aligned to a gene was divided by the total number of fragments that uniquely aligned to all genes and by the base number in the CDS of that gene. The FPKM method can eliminate the influence of different gene lengths and sequencing levels on the calculation of gene expression.

5.5 *In situ* hybridization

Two-color fluorescence *in situ* hybridization was performed according to previous works[56] for investigation of antennal localization of *CpomORco*, *CpomOR3* and *CpomOR3a* genes

in *C. pomonella* adults. Primers (**Supplementary Table 18**) were designed to synthesize Digoxigenin (Dig)- or Biotin (Bio)-labeled probes with an RNA labeling Kit version 12 (SP6/T7) (Roche, Mannheim, Germany), respectively. Male and female antennae of *C. pomonella* moths were dissected and embedded in JUNG tissue freezing medium (Leica, Nussloch, Germany) and stored at -80°C before sectioned (10 µm) with a freezing microtome (Leica, Nussloch, Germany). After fixation and hybridization, Digoxigenin was detected with anti-digoxigen (Roche) and Strepavidin-HRP (PerkinElmer, Boston, USA), and Biotin was detected with the TSA kit protocol (PerkinElmer). Prepared slides were analyzed with a Zeiss LSM710 Meta laser scanning microscope (Zeiss, Oberkochen, Germany). For better observation of both genes, we recorded Digoxigenin under green color and Biotin under purple, so that white signals could be observed in co-expressed somata. Twenty pairs of antennae in 4 technical replicates were used for each gender and data were processed with Zeiss LSM Image Browser 4.2 (Zeiss) and Adobe Illustrator.

6. Functional analysis of *CpomOR3a* and *CpomOR3b*

6.1 Insects

C. pomonella were reared in Chinese Academy of Inspection and Quarantine, Beijing, China. Larvae were fed on an artificial diet with 25 °C, 16:8 (L:D), 65% relative humidity. Pupae were placed in tube individually and selected by 2 days after eclosion. Male antennae were dissected and frozen in liquid nitrogen and then stored under -80 °C immediately until use.

6.2 Chemicals

(8E,10E)-Dodecadien-1-ol (Codlemone), ethyl-(2E,4Z)-decadienoate (Pear ester), Z8-dodecen-1-yl (Z8-12:OAc), E8-dodecen-1-yl (E8-12:OAc), Z8-dodecen-1-ol (Z8-12OH) were purchased from Nimrod Inc. (Changzhou, China). For two-electrode voltage clamp recordings, 1 M stock solution of each chemical was prepared in dimethyl sulfoxide, then

stored at -20 °C before use.

6.3 RNA extraction and cDNA synthesis

Male and female antennae were crushed in homogenizer and bathed in 1 ml of TriZol reagent (Invitrogen, Carlsbad, CA, USA). Then we prepared the extraction following the manufacturer's instruction. Total RNA was dissolved in nuclease-free water (Thermo Scientific). RNA quantity and quality were tested on a Nanodrop ND-1000 spectrophotometer (Nano-Drop products, Wilmington, DE, USA) and gel electrophoresis. RNA was treated with DNase I (Thermo Scientific) in order to remove residue of genome DNA before cDNA synthesis. The first-strand cDNA was synthesized from 2 μg of total RNA using the Revert Aid First Strand cDNA Synthesis Kit (Fermentas, Vilnius, Lithuania) and the cDNA product was either stored at -70 °C or used directly for PCR amplification.

6.4 Receptor expression in Xenopus oocytes and two-electrode voltage clamp recordings

The receptor expression and two-electrode voltage clamp recordings were performed according to the previous works[57] with some modifications. The full-length coding sequences of *CpomOR3a*, *CpomOR3b* and the co-receptor *CpomORco* (Genbank: JN836672.1) were amplified by PCR using the specific primers at both ends of ORFs, with carrying *Apa* I restriction site together with *Kozak* sequences in the forward primers and *Not* I restriction site in the reverse primers. The PCR products were digested with the both enzymes before ligation into PT7Ts vectors, which were previously linearized with the same enzymes. The cRNAs were synthesized from linearized vectors using mMESSAGE mMACHINE T7 Kit (Ambion, Austin, TX, USA). The cRNA mixture of 27.6 ng *CpomOrx* and 27.6 ng *CpomORco* was microinjected into the mature healthy oocytes (stage V–VII), which were previously treated with 2 mg/ml collagenase I in washing buffer (96 mM NaCl, 2 mM KCl, 5 mM $MgCl_2$, and 5 mM HEPES, pH 7.6) for 1-2 h at room temperature. After incubated for 4-7 days in incubation medium (1 x Ringer's buffer prepared with 0.8 mM

$CaCl_2$ in washing buffer at pH 7.6, 5% dialysed horse serum, 50 mg/ml tetracycline, 100 mg/ml streptomycin and 550 mg/ml sodium pyruvate) at 18 °C, the whole-cell currents against each chemical (10^{-4} M in 1 x Ringer's buffer) were recorded from the injected *Xenopus oocytes* using a OC-725C two-electrode voltage clamp (Warner Instruments, Hamden, CT, USA) at a holding potential of -80 mV. The data were acquired and analyzed with Digidata 1440A and Pclamp10.0 software (Axon Instruments Inc., Union City, CA, USA). Column charts were generated using GraphPad Prism 5 (GraphPad software, San Diego, CA, USA). Statistics were carried out using IBM SPSS Statistics 22.0.0 (SPSS, Chicago, IL, USA).

7. Genome re-sequencing

7.1 Insects for genome re-sequencing

To identify genetic changes conferring chemical insecticide resistance at genome level, two chemical insecticide resistant (Raz and Rv) and one chemical insecticide susceptible (S) strains provided by Dr. Pierre Franck and Dr. Myriam Siegwart of INRA (Avignon) were used in this study. Six third-instar larvae were randomly taken from each of the three strains, respectively. Rv and S were originated from the field a field population collected in 1995 using corrugated cardboard trapping strips in an apple orchard at Les Vignères (south-eastern France). The resistant strain Rv was derived from the field population by selection for the first 10 generations with increasing doses of deltamethrin. The progeny of isolated pairs was tested with discriminating doses of chemical in order to determine the parental genotype[58]. This procedure allowed the detection of susceptible adult pairs, whose progeny were used to build the susceptible strain (S). The second resistant strain, RA comes from a population collected in an apple orchard of Lerida region (Spain), where the organophosphate insecticide azinphos-methyl had become ineffective to control the codling moth before the sampling. Neonate progeny of the first 10 generations of this resistant population was selected by exposure to increasing concentrations of azinphos-methyl that induced 50% mortality. The S,

Raz and Rv strains were kept by mass rearing on an artificial diet[59] for more than twenty years. During the rearing period, S was never exposed to insecticides, whilst the Raz and Rv were submitted to selection pressure by spraying deltamethrin (2 mg L^{-1}) and azinphos-methyl (375 mg L^{-1}) on the surface of the artificial diet prior to penetration by newly hatched larvae[60], respectively.

7.2 Re-sequencing procedure

Total genomic DNA was isolated from the aforementioned 18 individuals, respectively. Genome of each individual was sequenced at the Shenzhen Millennium Spirit Technology Co., Ltd. The sequencing library is prepared by random fragmentation of the DNA or cDNA sample, followed by the protocol TruSeq Nano DNA Sample Preparation Guide, Part# 15041110 Rev. A. Adapter-ligated fragments are PCR amplified and gel purified. For cluster generation, the library is loaded into a flow cell where fragments are captured on a lawn of surface-bound oligos complementary to the library adapters. Each fragment is amplified into distinct, clonal clusters through bridge amplification. The templates are ready for sequencing when cluster generation is complete. Illumina SBS technology utilizes a proprietary reversible terminator-based method which detects single bases incorporated into DNA template strands. The Illumina Hiseq 4000 generates raw images using HCS (HiSeq Control Software v3.3) for system control and base calling through an integrated primary analysis software RTA (Real Time Analysis. v2.5.2), and the sequencing was following by the HiSeq 3000 4000 System User Guide Part # 15066496 Rev. A HCS 3.3.20. The BCL (base calls) binary is converted into FASTQ by illumina package bcl2fastq (V2.16.0.10, Illumina).

7.3 GWAS analysis of different strains which susceptible or resistance to insecticides

To identify variants between chemical insecticide samples and the respective susceptible samples. Variants calling and association analysis for all resistant-susceptible samples comparison (RA-SV and RD-SV for insecticide resistance) were performed (**Supplementary**

Fig. 16). First, genome sequencing data of all samples were subjected to control quality by FastQC[61] and Trimmomatic[62]. Then, the clean data of all samples were mapped to the genome assembly using BWA-mem[63] with default parameters. The overlapped reads in alignment were then removed by picard tools. Variants calling was performed between bam files of samples in each group by samtools[64] and bcftools[65]. Before the association analysis, variants stored in vcf files were filtered out by bcftools which removed variants with reads depth higher than 100 or quality less than 20% and by PLINK with the three thresholds: "--geno 0.05 --maf 0.01 --hwe 0.0001", which removed variants with missing genotype rates higher than 5%, minor allele frequency less than 1%, or Hardy-Weinberg equilibrium exact test p-value less than 0.001. Association analysis was performed between resistant strains and its corresponding susceptible strains by PLINK with the following parameters: --adjust --allow-extra-chr --allow-no-sex --assoc. Perl scripts were adopted to filter out the indel variants. To reduce the complexity of GWAS on identifying SNPs related to chemical insecticide resistance, we focused on the SNPs in 667 genes possibly involved chemical insecticide resistance from previous report[66]. Meanwhile, manhattan plot was drawn to visualize the SNPs located in cds regions in these 667 genes by qqman package of R[67].

8. SNPs validation and RNA interference

8.1 Insects & Chemicals

Ten individuals from each of the original three strains (S, Raz and Rv,) reared in INRA were used for SNP validation. Insects from a laboratory strain rearing in the Institute of Plant Protection, Chinese Academy of Agricultural Sciences was used for RNA interference. The strain originated from a field codling moth population collected in 2013 in Gansu Province of China, and was reared on artificial diet in the laboratory at 24± 1℃, 70% relative humidity and 16:8 h (L: D)

The deltamethrin (99.5% purity, Dr. Ehrenstorfer GmbH, Augsburg, Germany), azinphos-methyl (100% purity, AccuStandard, New Haven, CT, USA) and imidacloprid (99.0% purity,

Dr. Ehrenstorfer GmbH, Augsburg, Germany) was used for t bioassays after RNAi.

8.2 SNPs validation

Eleven SNPs which were significant different between the chemical insecticide resistant and susceptible sample were further confirmed in the individuals from the original strains by PCR. The PCR primers were designed according the sequences obtained. Ten individuals from S, Raz and Rv were used to check each of the SNPs, respectively (**Supplementary Table 21**).

8.3 siRNA injection

RNAi was used to analyze the role of insecticide detoxifying of a P450 genes (ID: CPOM05212.t1, referred as *CYP6B2*) with the same significant SNPs between chemical insecticide resistance and susceptible strains, as well as to test the function of *CpomOR3a/b*. Sequence-specific primers target the *CYP6B2* and *CpomOR3a/b* (**Supplementary Table 19**) were designed, and the siRNAs were chemically synthesized by Shanghai Gene Pharma (Shanghai, China) with 2' Fluoro dU modification to increase the stability of the siRNAs. The siGFP was synthesized and used as a control. The siRNAs and siGFP were dissolved with nuclease-free water to the concentration of 2 μg/μl and stored at − 80℃ until use.

For *CYP6B2* gene analyses, because all individuals of Raz and Rv strains were dead in 2018, we chose the Jiuquan strain which were used for *de novo* genome sequencing for function analysis. To knockdown *CYP6B2*, 0.5 μl siRNA was injected into the haemolymph of each forth-instar larva of Jiuquan strain using a microinjector (Femtojet Express, Eppendorf, Hamburg, Germany). The larvae injected with the same amount of siGFP and larvae had no injection were used as controls. Larvae were reared on artificial diet for 48 h post injection at 24± 1℃, 70% relative humidity and 16:8 h (L: D) until bioassay. For *CpomOR3a/b* gene functional test, 1 μl siRNA/siGFP was injected into the 9-day old pupae through the membrane. Moth will emerge from the survival pupae within 24 h post injection of *CpomOR3a/b*.

8.4 Quantitative PCR

To analyze the reduction of transcription levels of *CYP6B2* or *CpomOR3a/b*, total RNA was extracted from three survival larvae or adult heads of each treatment with the TRIzol reagent (Ambion, Thermo Fisher Scientific), respectively. The quantitative PCR (qPCR) reaction was performed with TransScript All-in-One First-Strand cDNA Synthesis SuperMix (TransGen Biotech, Beijing, China) according to the manufacturer's instruction using the ABI 7500 Real-Time PCR System (Applied Biosystems, CA, USA). The qPCR primers were designed from a different region of *CYP6B2* or *CpomOR3a/b* to those used for RNAi (**Supplementary Table 20**). The amplification steps for qPCR consisted of 95°C for 5 min, 40 cycles of denaturation at 95°C for 10 s, and extension at 60°C for 34 s to generate a melt curve. Three replications were carried out for each treatment. Data was calculated based on the $2^{-\Delta\Delta Ct}$ method with the mRNA relative expression normalized to *Cpomβ-tubulin*.

8.5 Electroantennogram tests with RNAi strains

Electroantennogram tests were adopted from previous works[68]. Antennae were processed following standard procedures by cutting both extremes of flagella and immediately mounted with two glass capillary Ag/AgCl electrodes containing Ringer solution[69]. Pear ester solutions were loaded on a filter paper piece at the same dosages with y-tube tests. At least 10 individuals were used as replicates for each chemical from each strain. Hexane was used as the carrier solvent and the blank control. Data were standardized following a standard protocol for EAG tests before compared between RNAi strains with siGFP strain by Student's *t* tests[70].

8.6 Y-tube olfactometer assays

Y-tube olfactometer indoor assays were adopted from our previous works on Lepidoptera adults[71]. The attractiveness of chemical volatiles was tested with 1 day old adults. Pear ester was used at the dose of 1 mg. The choice made within 5 min was recorded and at least 30

moths were tested in each pair. All tests were conducted at room temperature, i.e. 25 ± 2 °C, with constant purified and moistened air flow at a rate of 0.5 l/min, and odorant compounds were switched between the two arms every 5th test. *Chi*-square tests were used to compare the differences of counts' distributions between siGFP strain and each other injected strain.

8.7 Insecticide bioassay

After 48 h post injection, thirty survival larvae from each treatment were randomly collected for each bioassay, and thirty forth-instar native larvae without any injection were used as control. Three independent replicates were performed for each treatment and control. A droplet of 0.04μl insecticide solution was applied topically on the middle-abdomen notum of the larvae with a hand microapplicator (Burkard Manufacturing Co. Ltd, Richmansworth England)[60]. A droplet of 0.10 μl of the LC_{50} solution of azinphos methyl (103.50 mg/L) and deltamethrin (3.55 mg/L) and imidacloprid (35.35 mg/L) in distilled water containing 0.01% (v/v) Triton and 0.01% acetone was applied topically on the middle-abdomen notum of the larvae with a hand microapplicator (Burkard Manufacturing, Richmansworth, England). Control larvae were treated with distilled water containing 0.01% (v/v) Triton X-100 and 0.01% acetone. Survival rate of the treated larvae were assessed in 48 h after exposure to the chemicals. Survival rate data (percentage) were transformed using arcsine square-root transformation, and then subjected to ANOVA. All ANOVA was analyzed by Tukey's honest significant difference (HSD) using GraphPad Prism 6.0 (GraphPad Prism Software Inc., San Diego, USA). Counts were standardized into z-score before statistical analysis.

Supplementary Figures

Supplementary Figure 1

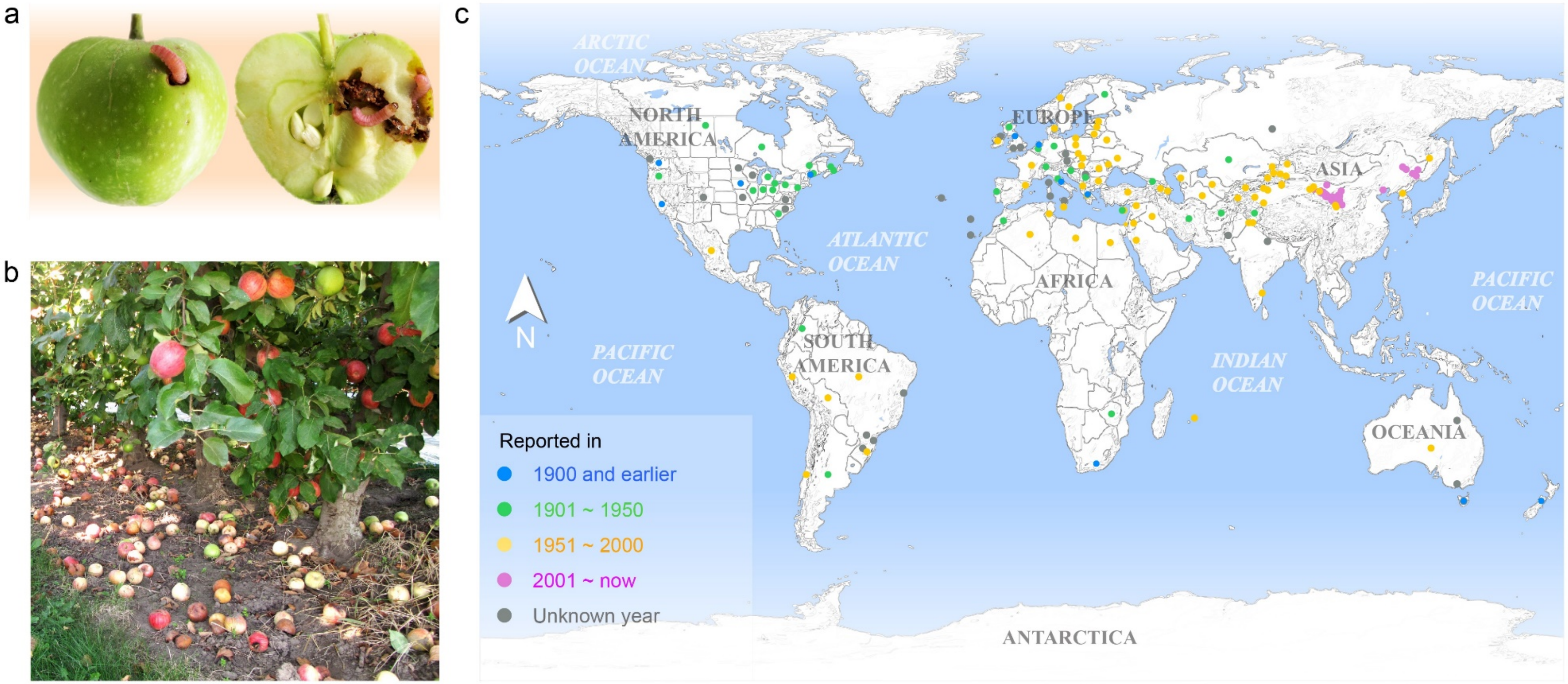

Supplementary Figure 1 Geographic distribution and damage of the codling moth, *Cydia pomonella*. (a) *C. pomonella* larva in apple fruit. **(b)** Damage caused by *C. pomonella* in apple orchard. **(c)** Distribution spots were located and timed according to published reports. *C. pomonella* was first recorded in Greece and Italy before the Christian era (red ellipse). Probably transported in packages containing infested apples and pears, it was then recorded present in

the Netherlands (recorded in 1635), United States (recorded in 1750), Graaff-Reinet in South Africa (recorded in 1855), Tasmania in Australia (recorded in 1855), New Zealand (recorded in 1874) and United Kingdom (recorded in 1897) (blue circles). From 1901 to 1950, *C. pomonella* widened its distribution in Europe and eastern North America, and started to enter South America (recorded in 1943) and western Asia (recorded in 1935) (green circles). Since then, it has accelerated its invasion. It is currently distributed in most areas in Europe, as well as in Asia, South America and northern Africa (yellow circles). Even under closely monitoring in China, newly detected occurring site of *C. pomonella* increased quickly in Gansu and Heilongjiang provinces where are the main producing area of apple (purple circles). Besides, there are some areas known for the occurrence of *C. pomonella,* but the first recorded year are unclear (grey circles).

Supplementary Figure 2

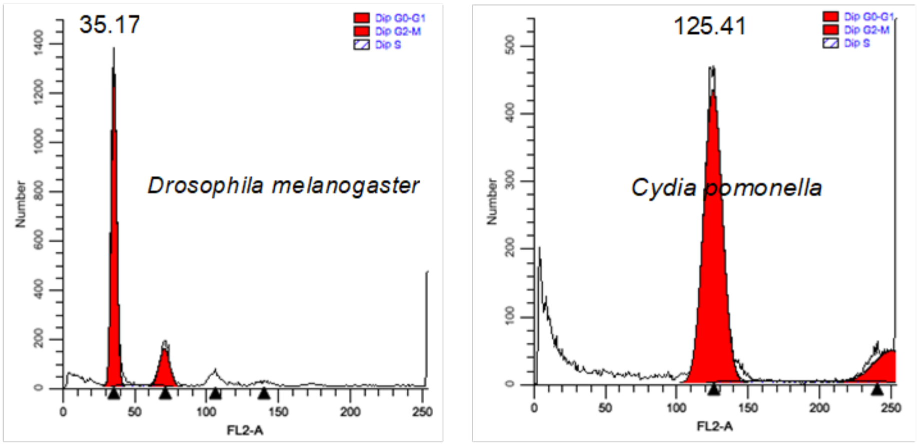

Supplementary Figure 2. Flow cytometry estimation of the genome size for the *Cydia pomonella*

Supplementary Figure 3

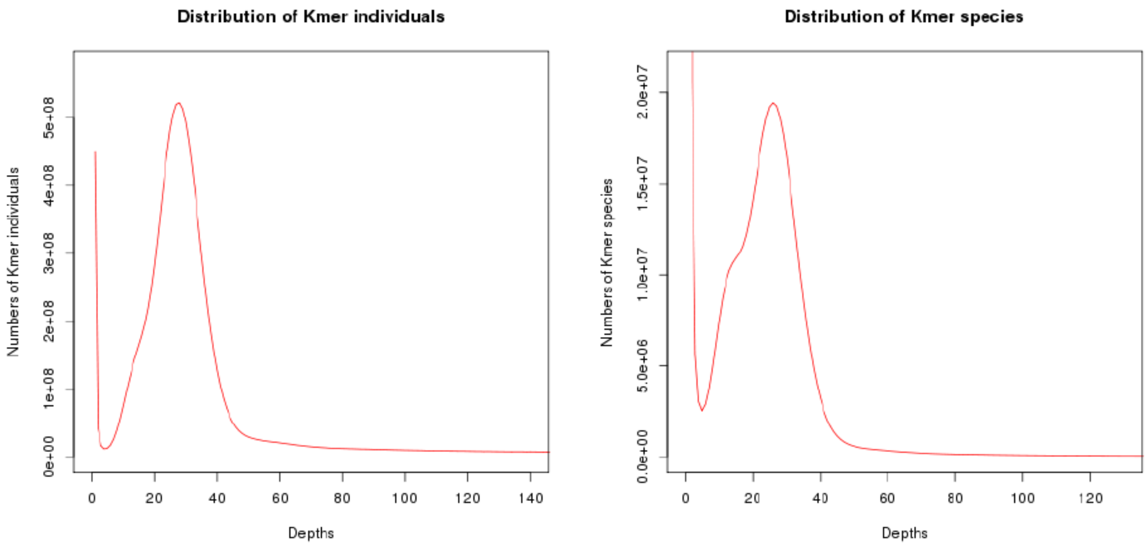

Supplementary Figure 3. The K-mer analysis of genome survey of *Cydia pomonella*. The genome survey sequencing data was used to count of k-mers in DNA by Using the software JELLYFISH with K=17.

Supplementary Figure 4

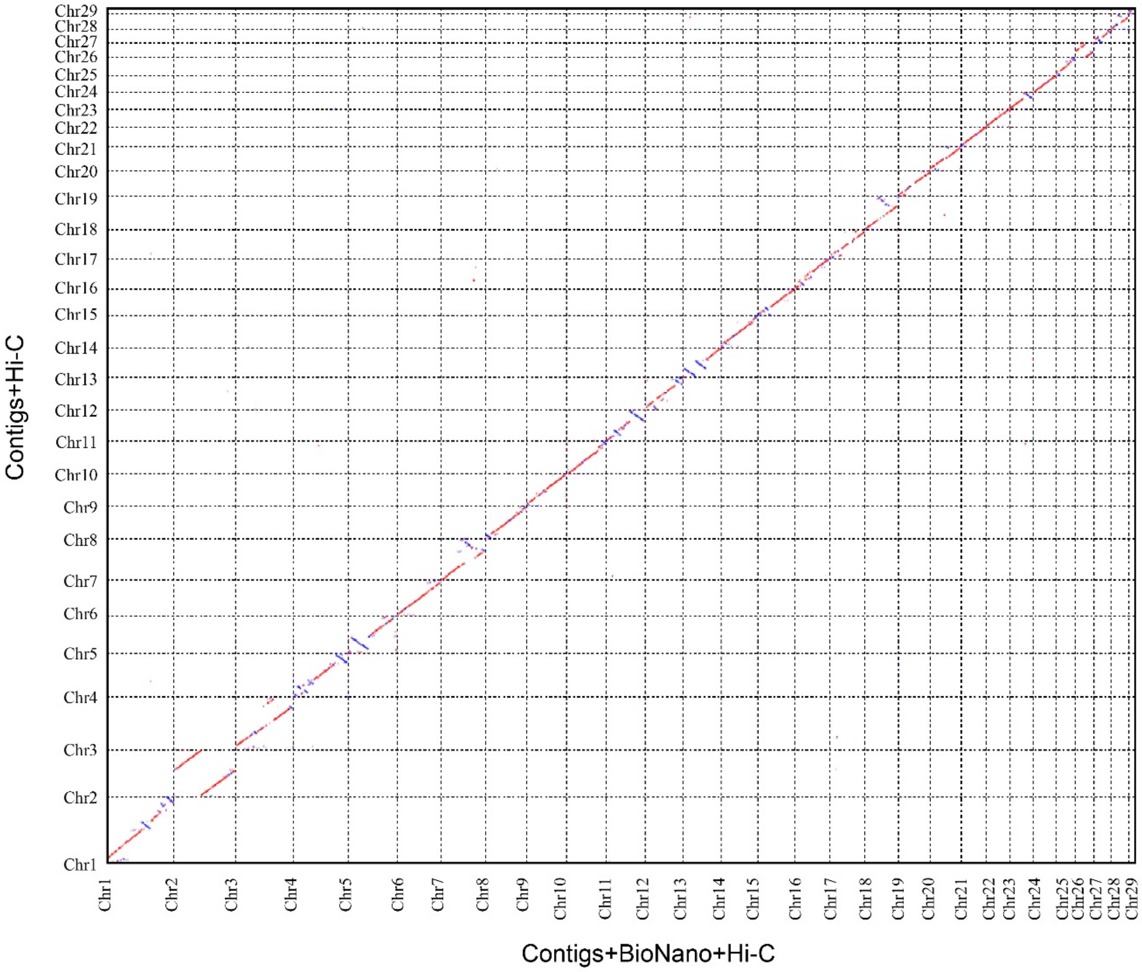

Supplementary Figure 4. The collinearity between the HiC super-scaffolds and BioNano-improved scaffolds of *Cydia pomonella*

Supplementary Figure 5

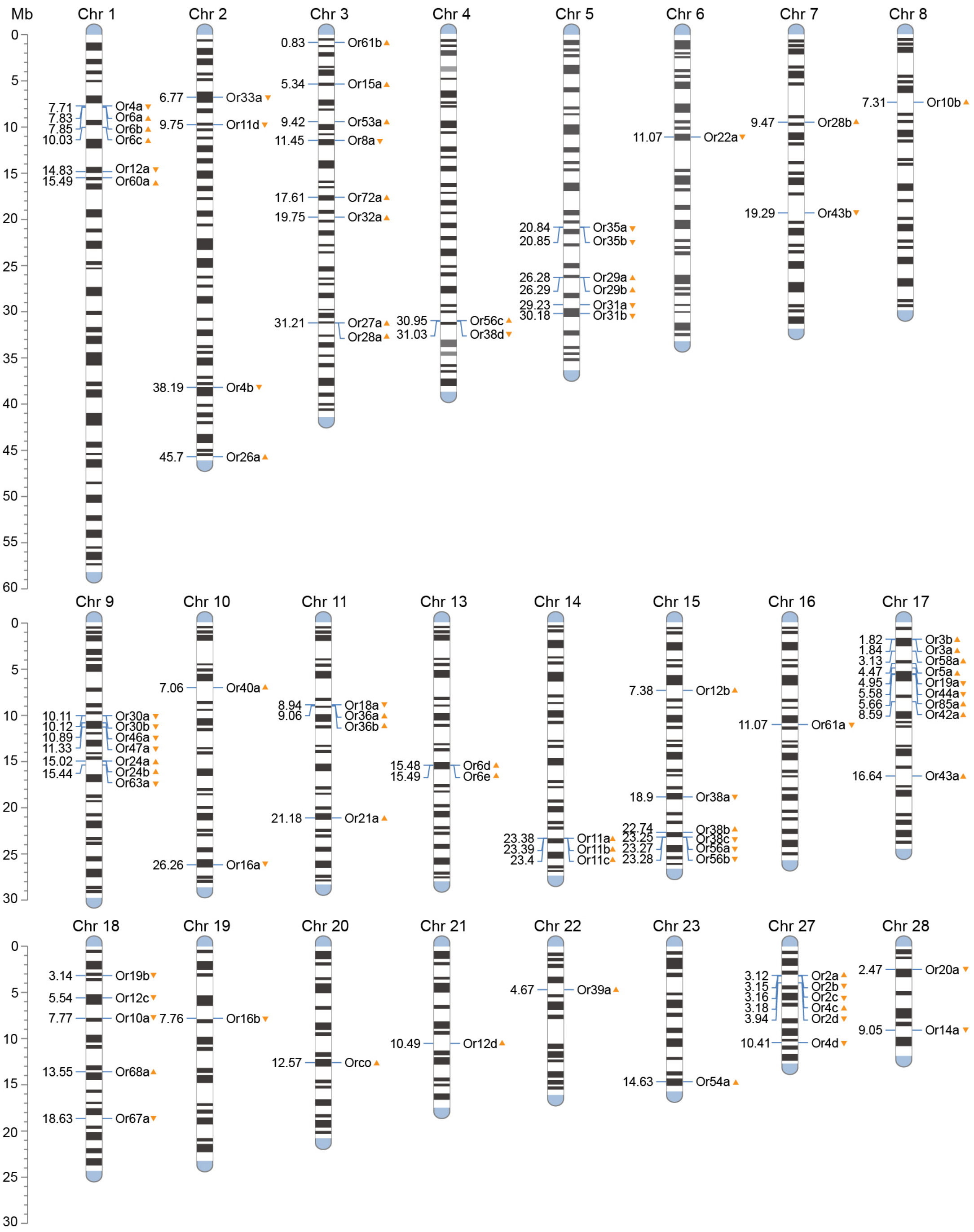

Supplementary Figure 5. The distribution of the OR genes on the chromosome of *Cydia pomonella*.

The upward orange arrow represents the gene on the positive chain and the downward arrow on the opposite chain.

Supplementary Figure 6

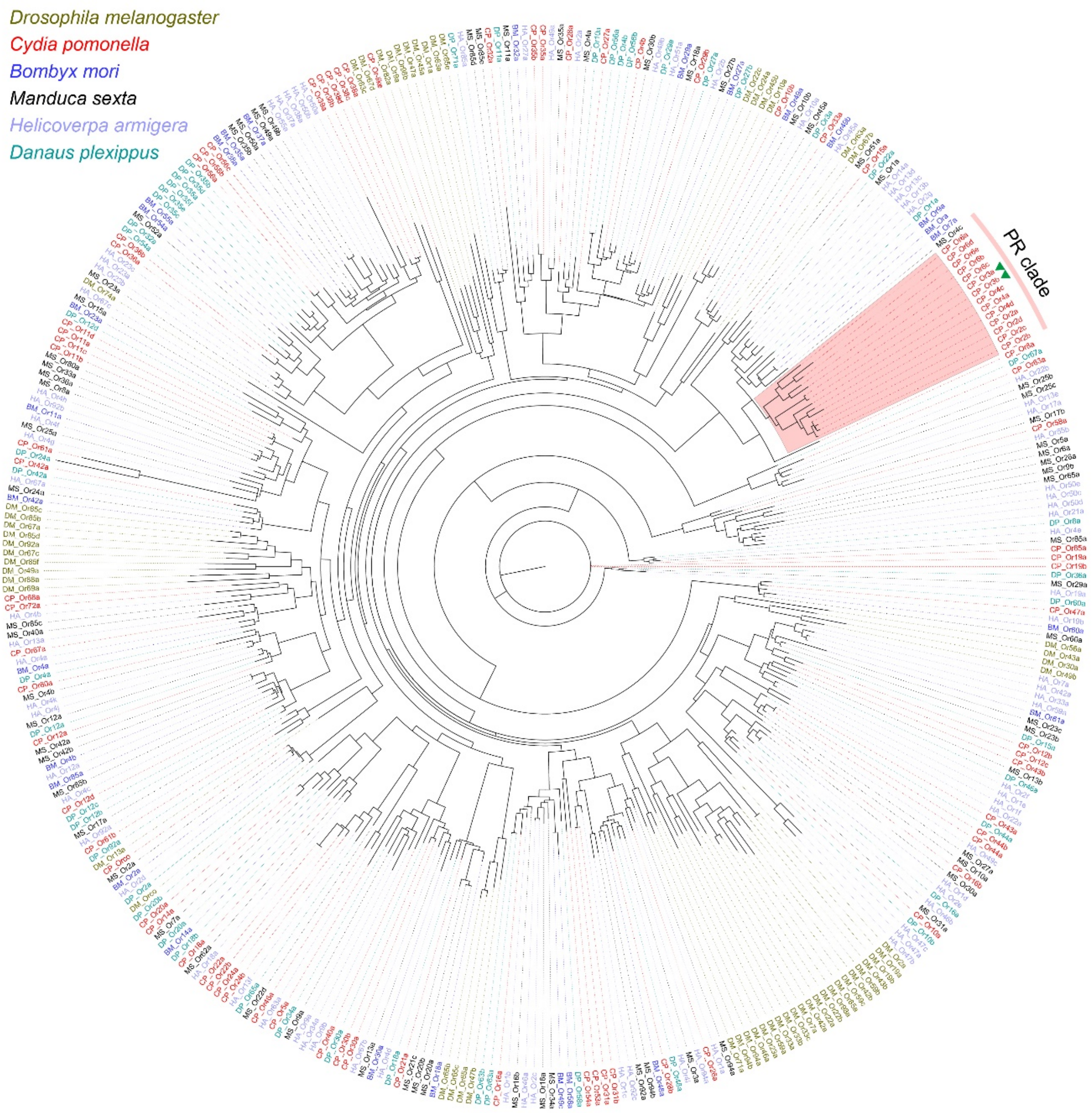

Supplementary Figure 6. Maximum likelihood phylogenetic tree established from candidate *CpomOR* sequences of *Cydia pomonella* and *OR* sequences from other 9 insect species. The evolutionary history was inferred using the maximum likelihood method. The analysis involved 368 amino acid sequences. All positions containing gaps and missing data were eliminated. Meanwhile, the best substitution model "LG+F+G4" was chosen. Phylogenetic tree was conducted in RAxML v8.2.10. Maximum-likelihood tree revealed a strong expansion of the *OR* genes in the genome of *Cydia*

pomonella (Cpom). Included are all *OR* genes identified in the genomes of five lepidopteran insects *Bombyx mori* (Bmor), *C. pomonella*, *Danaus plexippus* (Dple), *Helicoverpa armigera* (Harm) and *Manduca sexta* (Msex) as well as a model insect *Drosophila melanogaster* (Dmel). The OR genes of *C. pomonella* are highlighted by red words. Pink region indicates codlemone and pear ester receptor clade. Arrows indicates *CpomOR3a* and *CpomOR3b*.

Supplementary Figure 7

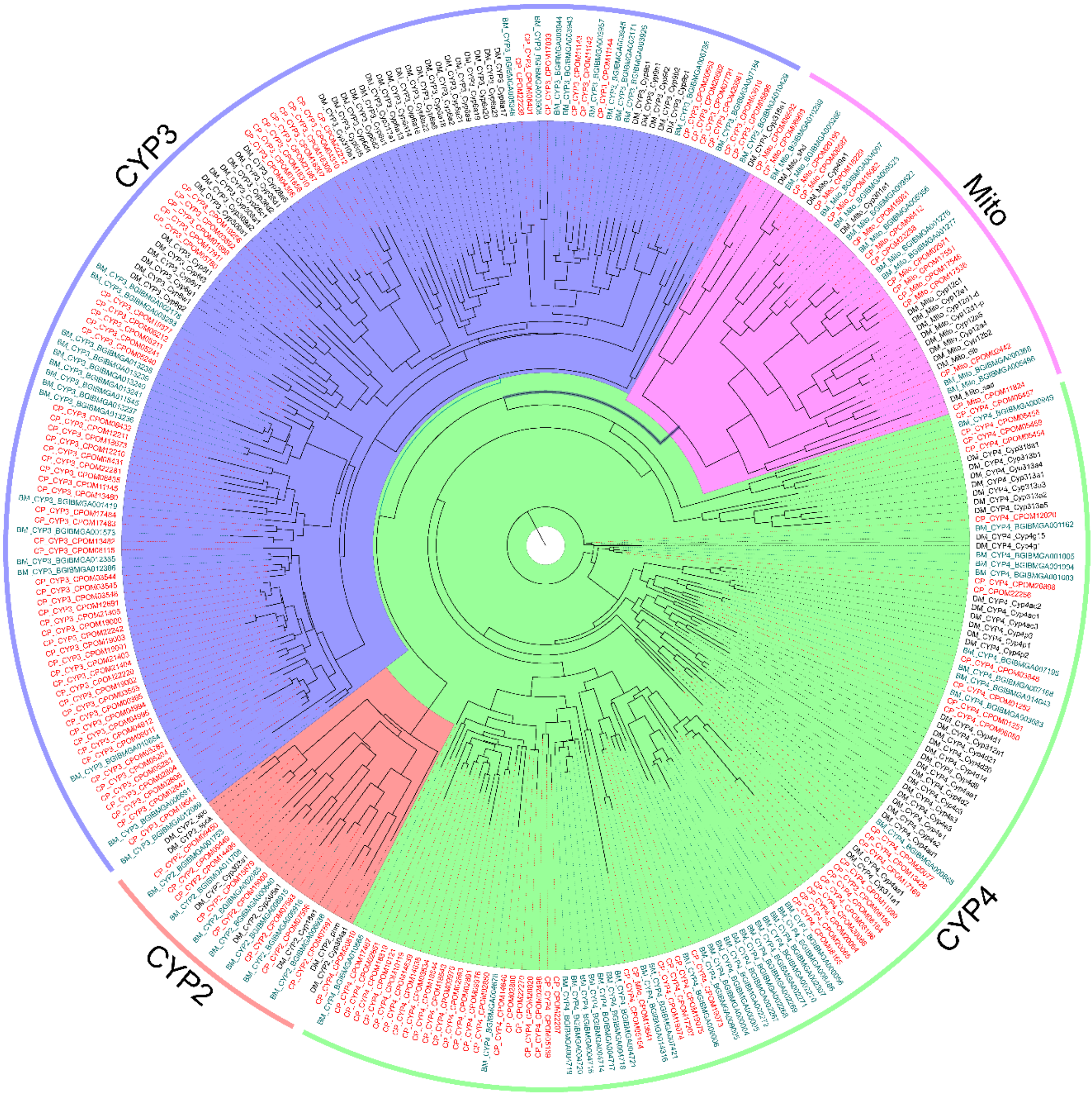

Supplementary Figure 7. Phylogenetic trees of *Cydia pomonella* cytochrome P450 (P450) gene family with other insects. Bmor, *Bombyx mori*; Cpom, *Cydia pomonella*; Dmel, *Drosophila melanogaster*. The trees were constructed using maximum likelihood (ML) method by RAxML software and optimized by Figtree software.

Supplementary Figure 8

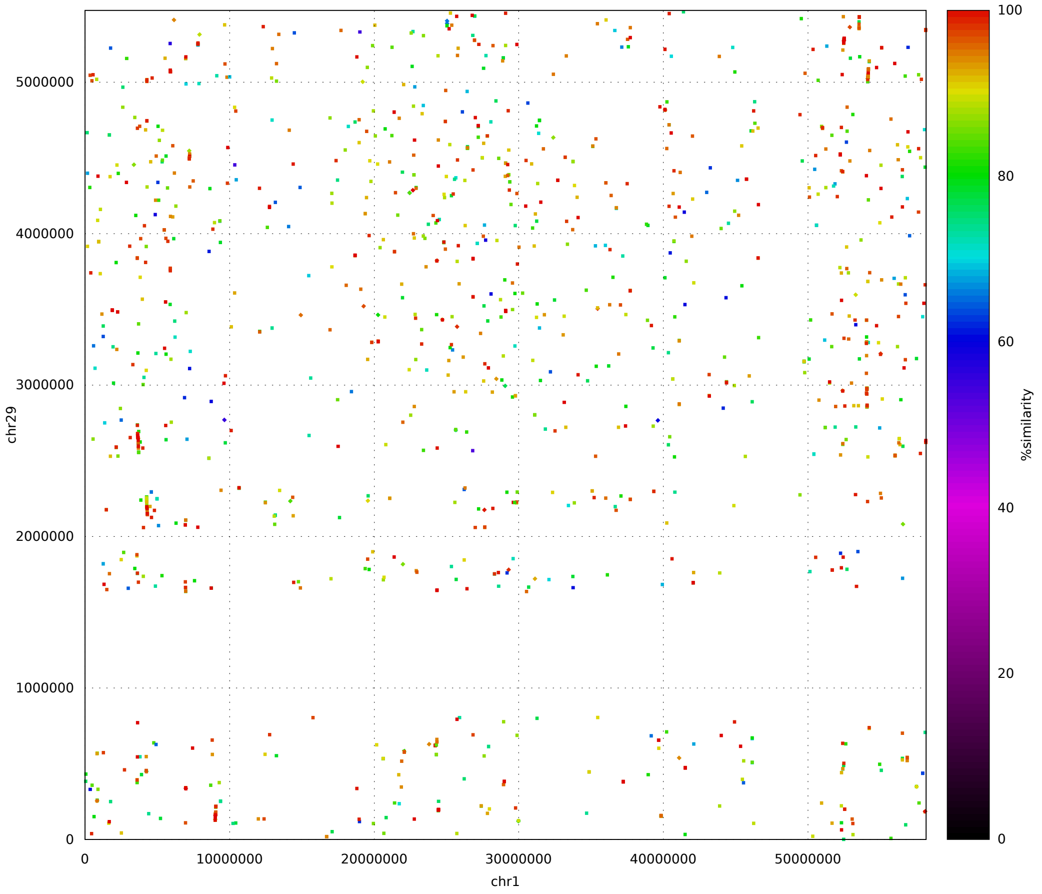

Supplementary Figure 8. **Translated alignment of chr29 (W) against chr1 (Z) of *Cydia pomonella*.** This figure is representative of various approaches to whole-scaffold alignments between the Z and W scaffolds. Depicted are the results of PROmer.

Supplementary Figure 9

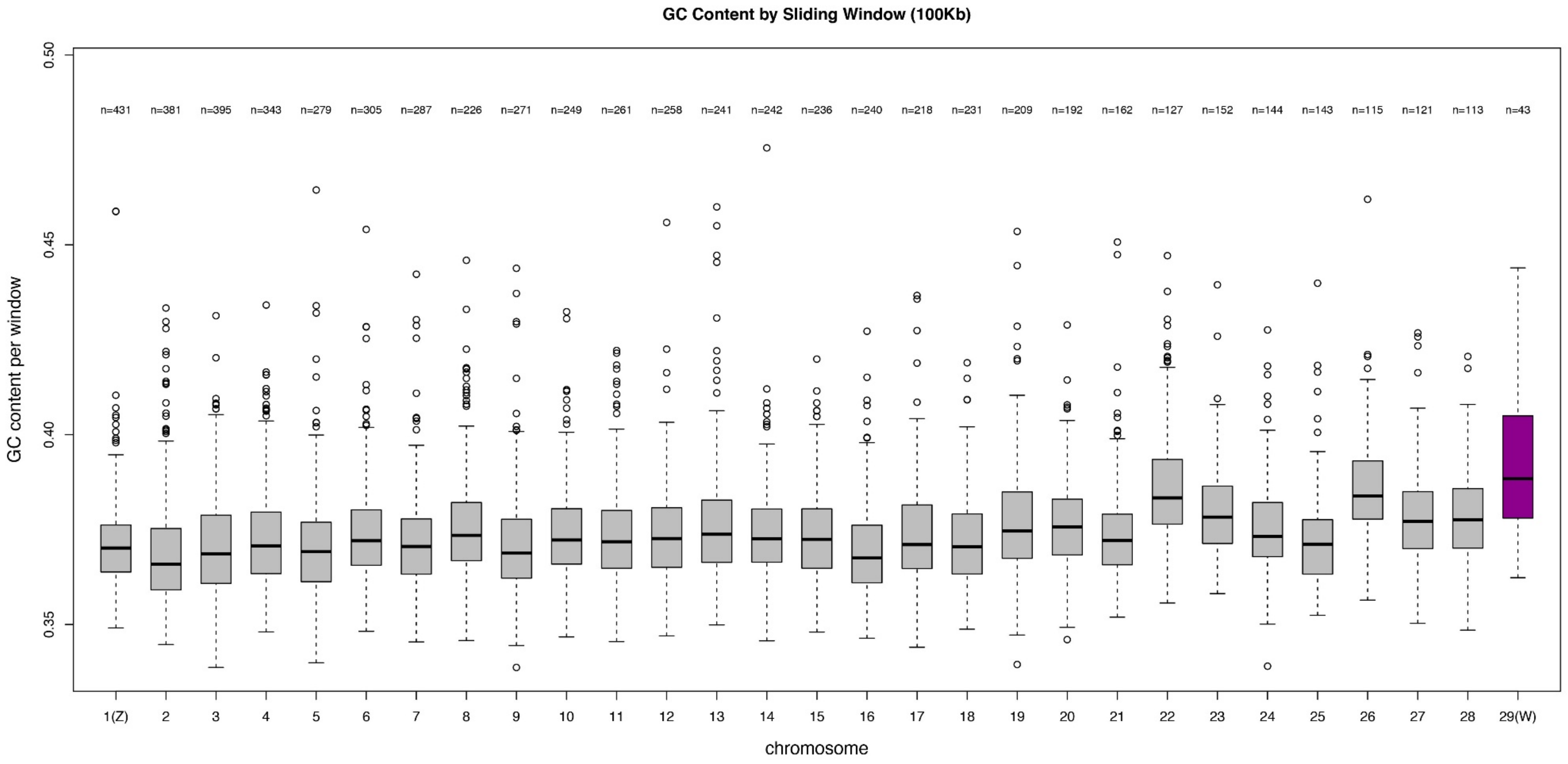

Supplementary Figure 9. GC content by sliding window (100Kb) across the chromosomes of *Cydia pomonella*.

Supplementary Figure 10

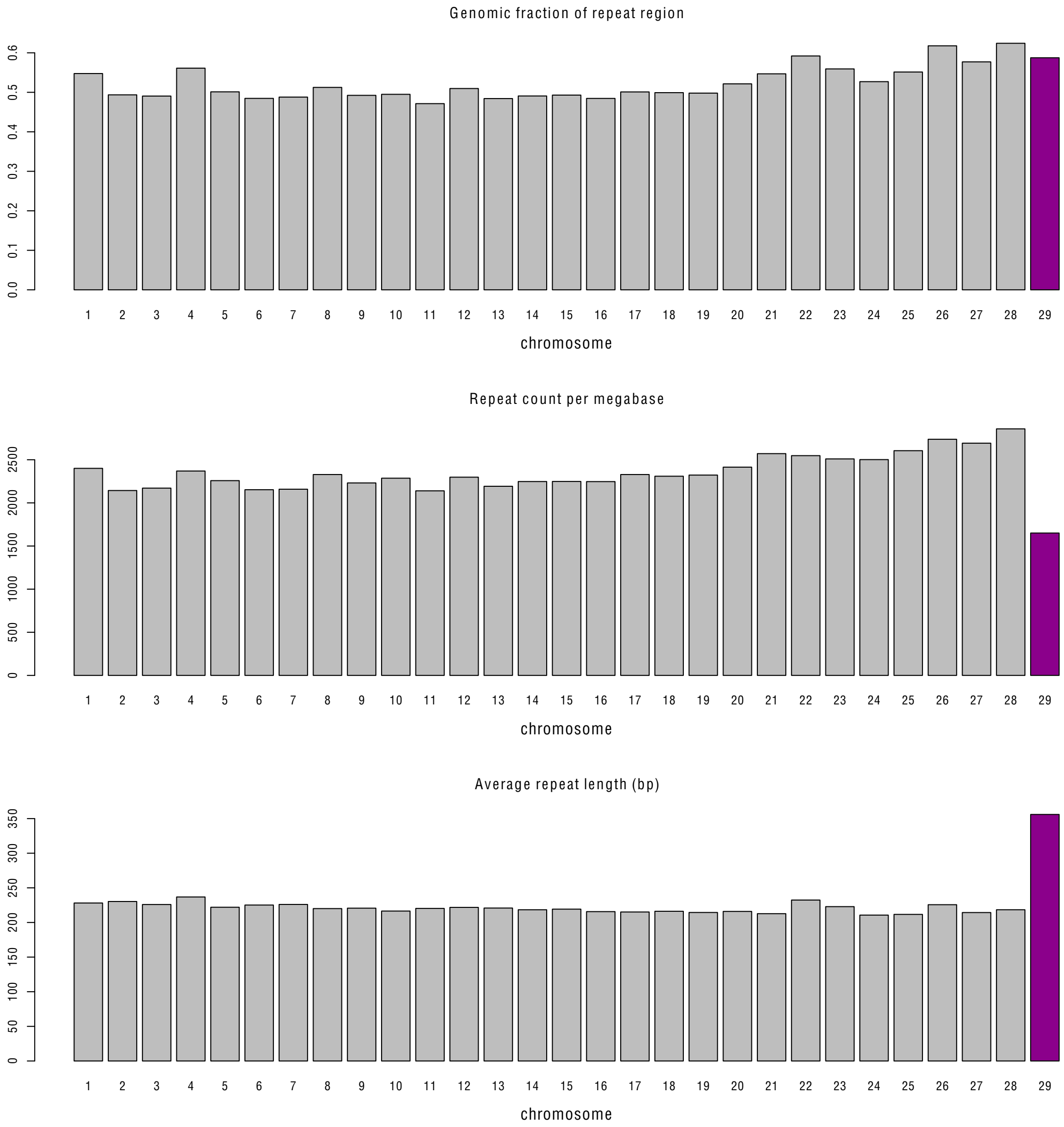

Supplementary Figure 10. Characteristics of repeat content across the chromosomes of *Cydia pomonella*. Chr29 corresponds to the W, in purple.

Supplementary Figure 11

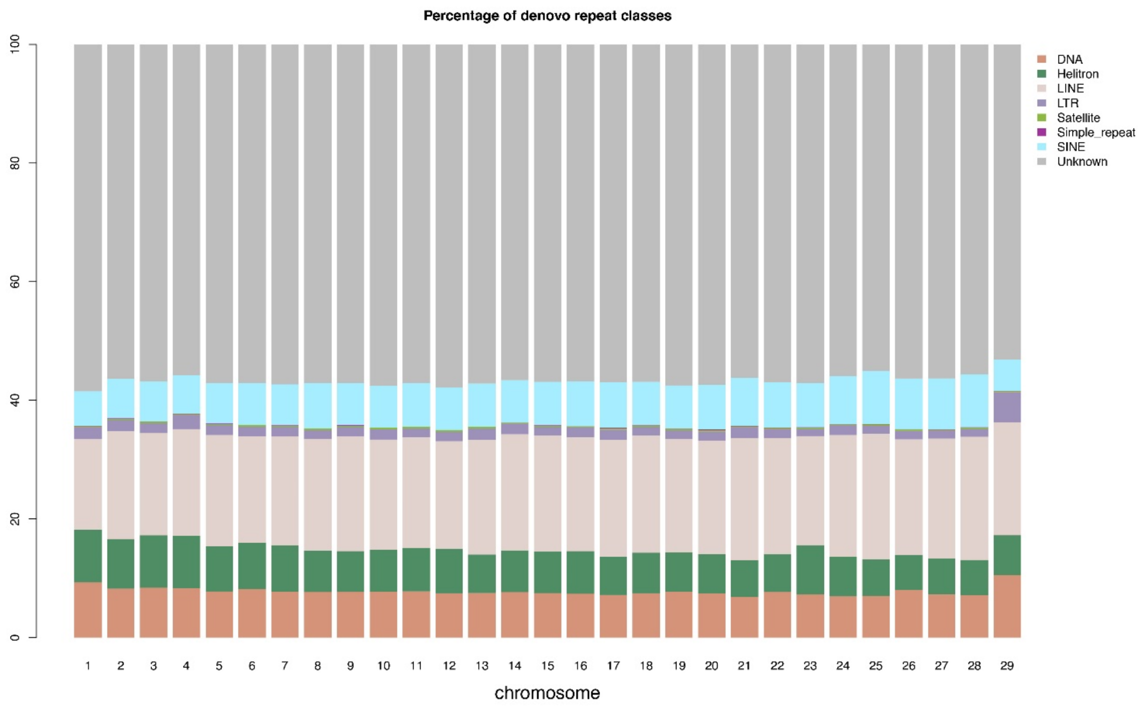

Supplementary Figure 11. Percentage of *de novo* repeat classes across the chromosomes of *Cydia pomonella*.

Supplementary Figure 12

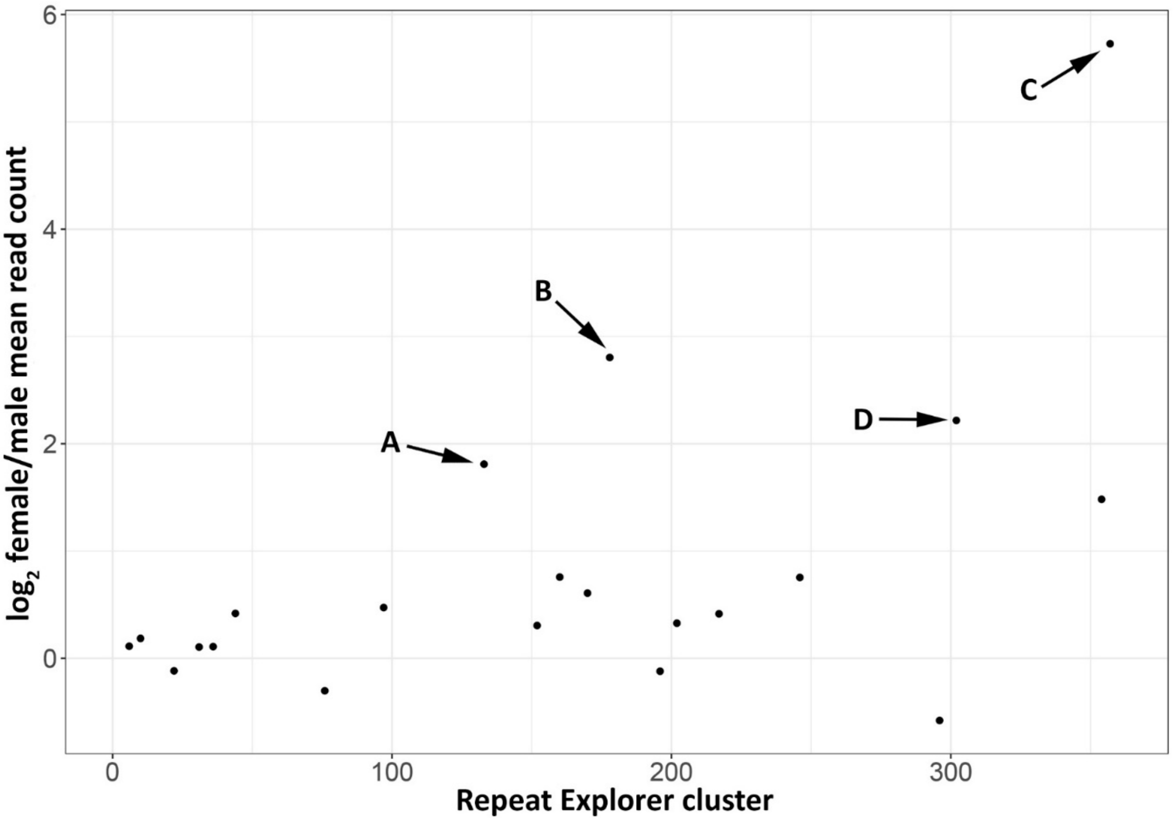

Supplementary Figure 12. Representative results of Repeat Explorer analysis indicating repeats with female-specific enrichment of *Cydia pomonella*. Each repeat is represented by one or more clusters. Clusters without significant difference between females and males (t-test; $P \geqslant 0.05$) were omitted. Arrows indicate clusters corresponding to four repeats (A, B, C, and D; see Supplementary Table 17 for details) with at least two times female enrichment in more than half of performed Repeat Explorer analysis.

Supplementary Figure 13

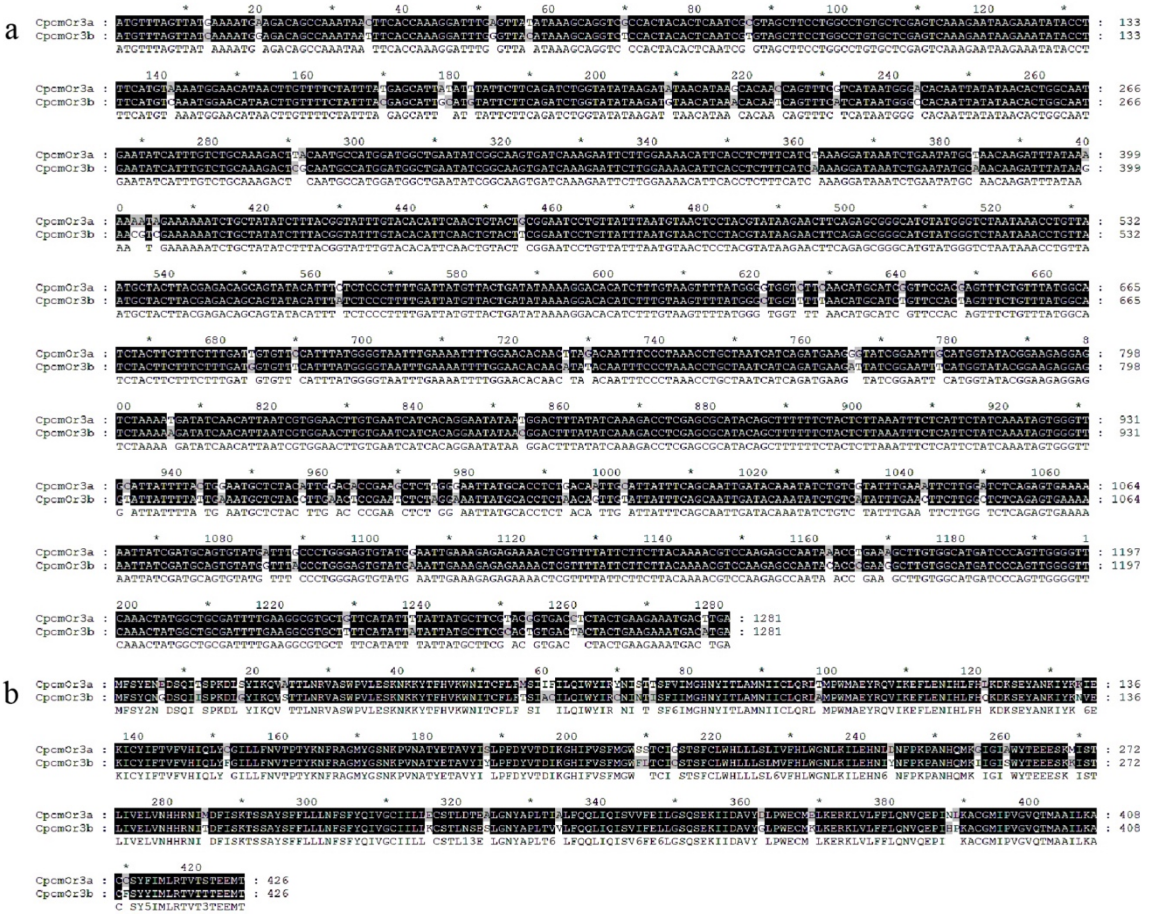

Supplementary Figure 13. Multiple sequence alignment of *Cydia pomonella OR3a* and *OR3b*. Nucleotide **(A)** and amino acid **(B)** sequence similarity of *CpomOR3a* and *CpomOR3b* was shown. Conserved residues between *CpomOR3a* and *CpomOR3b* were indicated with black blocks. The sequence identities of nucleotide sequences and amino acid sequences were 94% and 89%, respectively.

Supplementary Figure 14

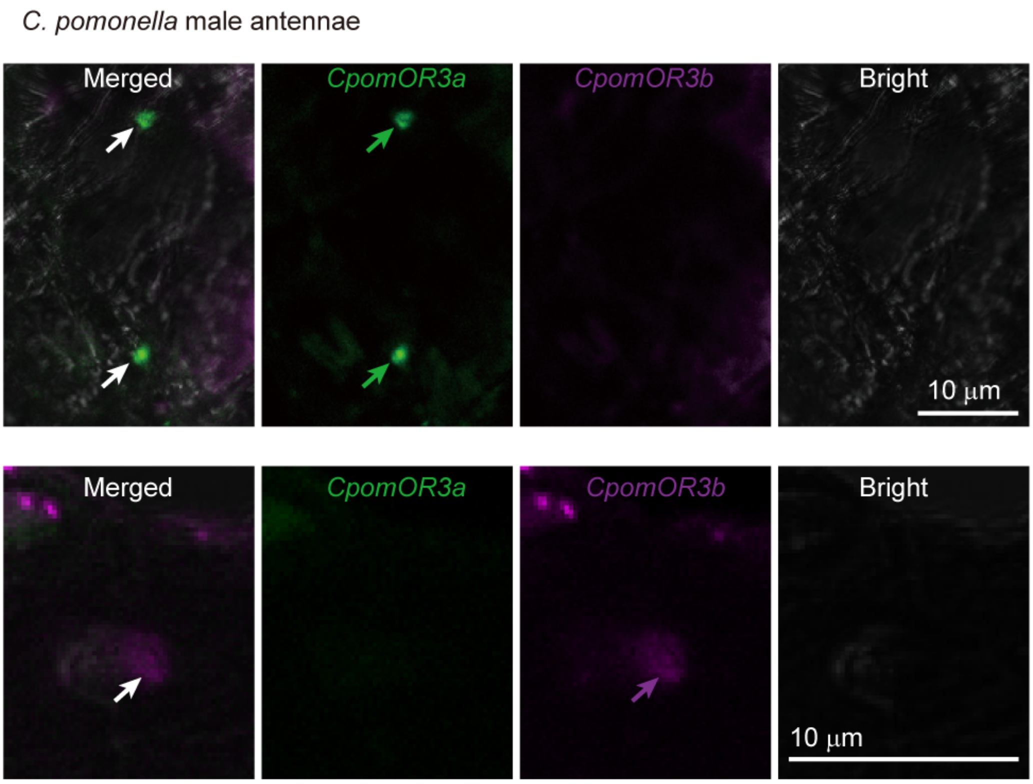

Supplementary Figure 14. Expression patterns of *CpomOR3a* and *CpomOR3b* in male *Cydia pomonella* antennae. *CpomOR3a* was labeled with Digoxigenin in green and *CpomOR3b* was labeled with Biotin in purple. Up: Single expression of *CpomOR3a*; down: Single expression of *CpomOR3b.* Source data are provided as a Source Data file

Supplementary Figure 15

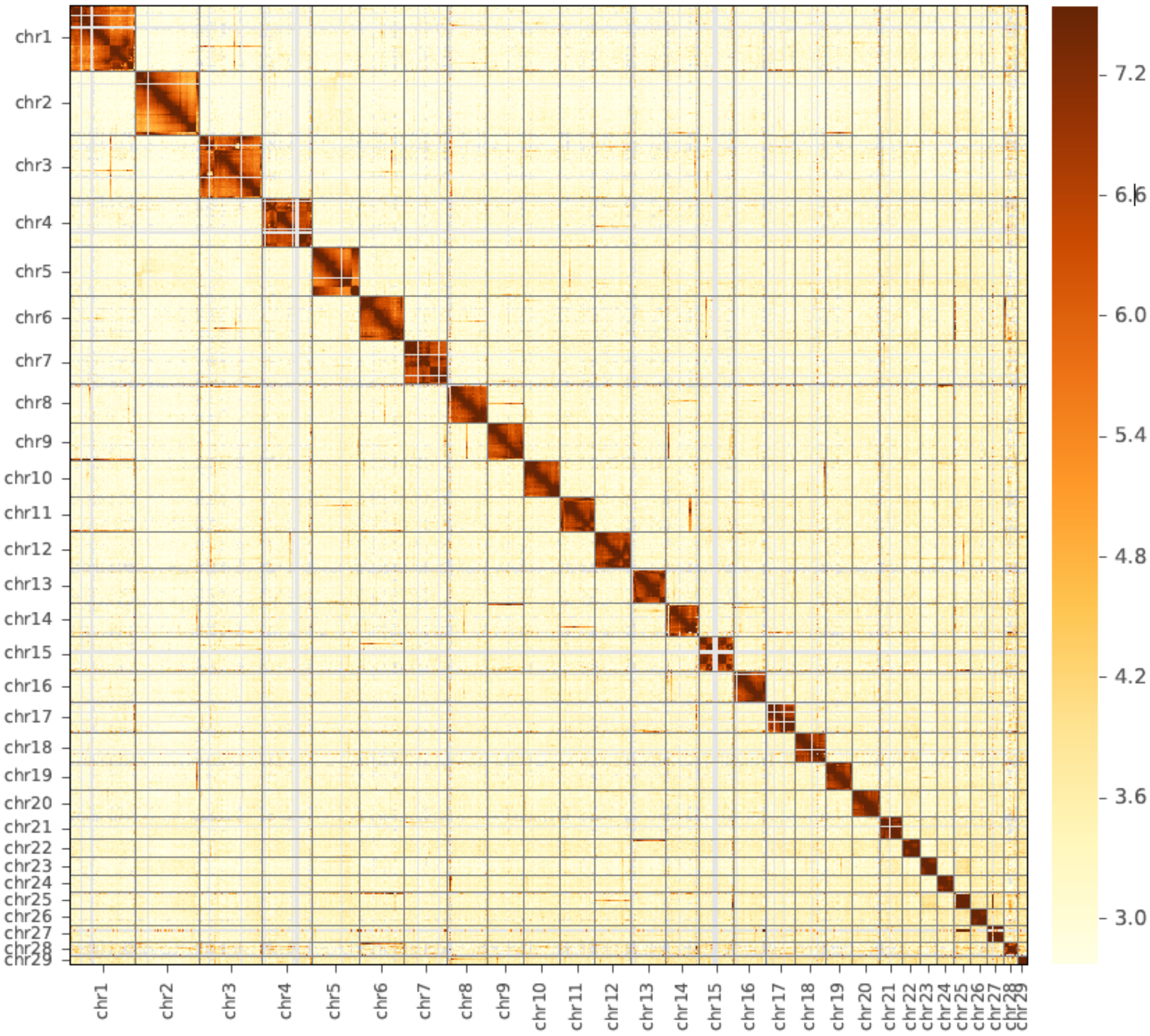

Supplementary Figure 15. Genome-wide all-by-all HiC interaction (Cited in Online methods).

Supplementary Figure 16

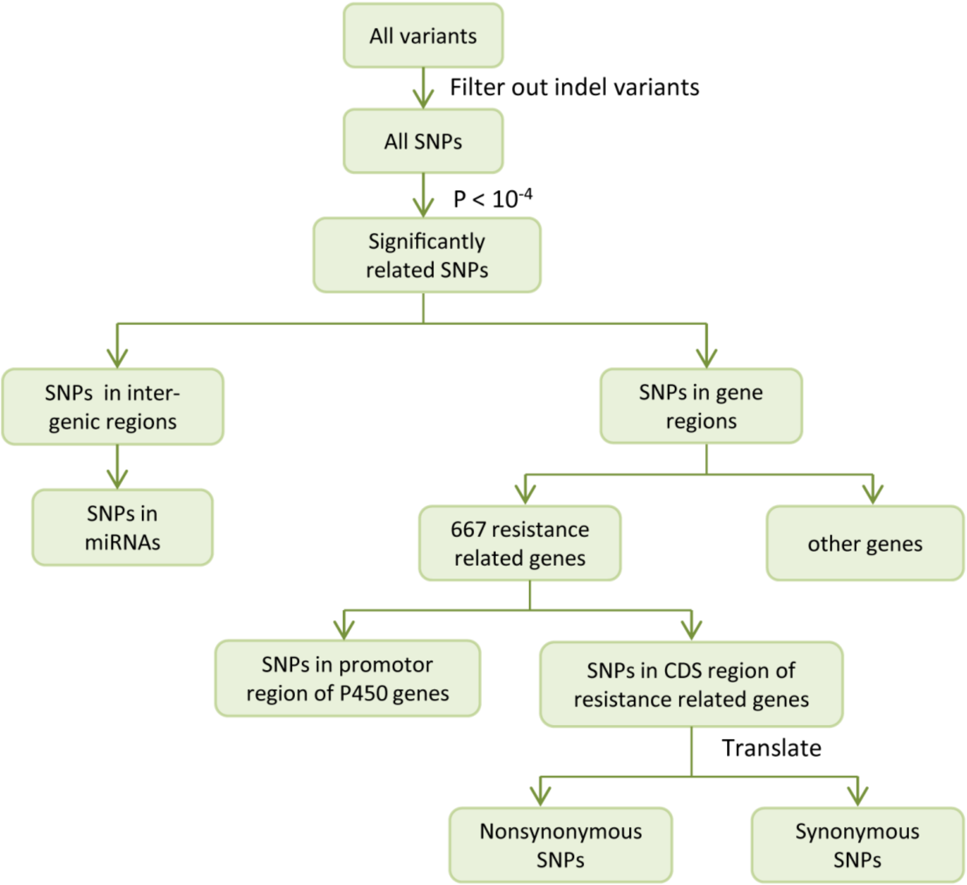

Supplementary Figure 16. GWAS analysis workflow to select the SNPs in potential regions which was reported to be associated with insecticide resistance (Cited in Online methods).

Supplementary Tables

Supplementary Table 1. Geographic distribution records of the codling moth, *Cydia pomonella*

Continent	Country and corresponding area	Distribution	Year	Reference
Europe	Greek	Origin	B.C.371	(Tadi′, C. & Milorad, D. 1963)[72]
Europe	Italy	Present	B.C.200	(Slingerland, M. V. 1898)[73]
Europe	Netherlands	Present	1635	(Slingerland, M. V. 1898)[73]
North America	USA, New England	Restricted distribution	1750	(Slingerland, M. V. 1898)[73]
North America	USA, California	Present	1819	(Simpson C. B. 1903)[74]
North America	USA, Central New York	Present	1840	(Simpson C. B. 1903)[74]
Oceania	Australia	Present	1855	(Simpson C. B. 1903)[74]
North America	USA, Iowa	Present	1860	(Simpson C. B. 1903)[74]
Oceania	Australia, Tasmania	Present	1861	(Simpson C. B. 1903)[74]
North America	USA, California, Sacramento	Present	1872	(Simpson C. B. 1903)[74]
Oceania	New Zealand	Widespread	1874	(Simpson C. B. 1903)[74]
North America	USA, Utah	Present	1874	(Simpson C. B. 1902)[75]
North America	USA, Washington	Present	1880	(Simpson C. B. 1902)[75]
Africa	South Africa, Graaff-Reinet	Present	1885	(Lounsbury, C. P. 1898)[76]
North America	Idaho, Boise & Clearwater Valley	Present	1887	(Simpson, C. B. 1902)[75]
South America	Brazil	Present	Before 1891	(Simpson, C. B. 1903)[74]
North America	Canada, Ottawa	Present	1885	(Slingerland, M. V. 1898)[73]
Europe	UK, England and Wales	Widespread	1897	(UK, Ministry of Agriculture and Fisheries, 1918)[77]
North America	USA, Washington, Pullman	Present	1898	(Simpson, C. B. 1903)[74]
Europe	Russia, Moscow	Present	1899	(Simpson, C. B. 1903)[74]
North America	USA, life zones in Idaho	Present	1901	(Simpson, C. B. 1902)[75]
North America	USA, Western Oregon	Present	1901	(Simpson, C. B. 1902)[75]
North America	USA, Colorado, Fort Collins	Present	1902	(Simpson C. B. 1903)[74]
North America	Canada, Nova Scotia	Present	Before 1903	(Simpson C. B. 1903)[74]
North America	USA, Northern Maine	Present	Before 1903	(Simpson C. B. 1903)[74]
North America	USA, Northern Michigan	Present	Before 1903	(Simpson C. B. 1903)[74]
North America	USA, Alleghanian orogeny (Allegheny Mountains)	Present	Before 1903	(Simpson C. B. 1903)[74]
North America	USA, Montana, Helena	Present	Before 1903	(Simpson C. B. 1903)[74]
North America	USA, Oregon, Hood River Valley	Present	1903	(Simpson C. B. 1903)[74]
North America	Canada, British Columbia, Victoria	Present	1905	(Evans, H. H. 1921)[78]
Europe	Croatia	Present	1913	(Woodworth, C. W. 1913)[79]
North America	USA, Arkansas	Present	Before 1915	(Sanderson E. D. *et al.* 1915)[80]
North America	USA, New Mexico	Present	Before 1915	(Sanderson E. D. *et al.* 1915)[80]
North America	USA, Georgia	Present	Before 1915	(Sanderson E. D. *et al.* 1915)[80]
North America	Canada, Ontario	Present	1915	(Hall, J. A. 1929)[81]
North America	USA, Massachusetts	Present	1919	(Fernald 1919)[82]
Europe	Germany	Present	1920	(Lehmann 1922)[83]
Africa	Zimbabwe	Present	1922	(Jack, R.W. 1922)[84]
Europe	Portugal	Present	1924	(Washington, D. C. 1925)[85]
Europe	Cyprus	Present	1925	(Wilkinson, D. S. 1925)[86]
Europe	UK, Scotland	Present	1925	(Macdougall, R. S. 1926)[87]
North America	USA, Ohio	Present	1925	(Cutright, C. R. 1937)[88]
North America	USA, Pennsylvania	Present	1903	(Simpson, C. B. 1903)[74]
South America	Argentina	Present	1925	(Xu, J. *et al.* 2015)[89]

Asia	Japan	Present	1927	(Khajuria, D. R. *et al.* 1986)[90]
Oceania	Australia, New South Wales, Bathurst	Present	1928	(Allman, 1928)[91]
North America	USA, Illinois	Present	1929	(Flint, W. P. 1929)[92]
North America	USA, South Carolina	Present	1930	(Anonymous 1930)[93]
Europe	Finland	Widespread	1932	(Vappula, N. A. 1935)[94]
Europe	Bulgaria	Present	1932	(Askew, R. R. 1964)[95]
Africa	Morocco	Present	1933	(Iraqui & Hmimina, 2016)[96]
Europe	Switzerland	Present	1933	(Staehelin, M. & Bovey, P. 1940)[97]
North America	USA, Indiana	Present	1934	(Steiner, L. F.)[98]
Asia	Iran	Present	1935	(Xu, J. *et al.* 2015)[89]
Asia	Afghanistan	Present	1935	(S.M.DAS. 1938)[99]
Asia	India, Ladakh	Restricted distribution	1938	(Janjua, N. A. 1938)[100]
South America	Uruguay	Present	1943	(Xu, J. *et al.* 2015)[89]
Europe	Serbia	Present	1947	(Lekic, M. B. 1950)[101]
North America	Canada, New Brunswick	Present	1948	EPPO, 2014
North America	Canada, Prince Edward Island	Present	1948	EPPO, 2014
North America	Canada, Quebec	Present	1948	EPPO, 2014
Europe	Belgium	Present	1949	(Paternotte, E. 1989)[102]
Asia	Kazakhstan	Present	1950	(Xu, J. *et al.* 2015)[89]
Asia	China, Xinjiang, Kuerle (Korla)	Present	1953	(Zhang, X. Z. 1957)[103]
Asia	China, Xinjiang, Wulumuqi (Urumqi)	Present	1953	(Zhang, X. Z. 1957)[103]
Asia	China, Xinjiang, Yili	Present	1953	(Zhang, X. Z. 1957)[103]
Asia	China, Xinjiang, Tacheng	Present	1953	(Zhang, X. Z. 1957)[103]
Asia	China, Xinjiang, Aletai (Altay)	Present	1953	(Xu, J. *et al.* 2015)[89]
Asia	China, Xinjiang, Hetian (Hotan)	Present	1953	(Zhang, X. Z. 1957)[103]
Asia	China, Xinjiang, Kashi	Present	1953	(Zhang, X. Z. 1957)[103]
Asia	China, Xinjiang, Akesu (Aksu)	Present	1953	(Zhang, X. Z. 1957)[103]
Asia	China, Xinjiang, Alaer (Alar/Alear)	Present	1953	(Wang, L. *et al.* 2011)[104]
Asia	China, Xinjiang, Shihezi	Present	1953	(Xu, J. *et al.* 2015)[89]
Asia	China, Xinjiang, Jinghe	Present	1953	(Zhu, H. Y. *et al.* 2017)[105]
Asia	China, Xinjiang, Wulumuqi, Manasi (Manas)	Present	1953	(Zhu, H. Y. *et al.* 2017)[105]
Asia	China, Xinjiang, Wulumuqi, Tulufan (Turpan)	Present	1953	(Zhang, X. Z. 1957)[103]
Asia	China, Xinjiang, Wulumuqi, Shanshan	Present	1953	(Zhang, X. Z. 1957)[103]
Asia	China, Xinjiang, Luntai	Present	1953	(Zhang, X. Z. 1957)[103]
Asia	China, Xinjiang, Kuche (Kuqa)	Present	1953	(Zhang, X. Z. 1957)[103]
Asia	China, Xinjiang, Shaya (Xayar)	Present	1953	(Zhang, X. Z. 1957)[103]
Asia	China, Xinjiang, Xinhe	Present	1953	(Zhang, X. Z. 1957)[103]
Asia	China, Xinjiang, Baicheng	Present	1953	(Zhang, X. Z. 1957)[103]
Asia	China, Xinjiang, Wensu	Present	1953	(Zhang, X. Z. 1957)[103]
Asia	China, Xinjiang, Jiashi	Present	1953	(Zhang, X. Z. 1957)[103]
Asia	China, Xinjiang, Shache	Present	1953	(Zhang, X. Z. 1957)[103]
Asia	China, Xinjiang, Moyu	Present	1953	(Zhang, X. Z. 1957)[103]
Asia	China, Xinjiang, Yining	Present	1953	(Xu, J. *et al.* 2015)[89]
Asia	Syria	Present	1954	(Mansour, M. 2010)[106]
Asia	China, Xinjiang, Emin	Present	1953 ~1957	(Xu, J. *et al.* 2015)[89]
Asia	China, Xinjiang, Gongliu	Present	1953 ~1957	(Xu, J. *et al.* 2015)[89]
Asia	China, Xinjiang, Hoboksar	Present	1953 ~1957	(Xu, J. *et al.* 2015)[89]
Asia	China, Xinjiang, Horgas	Present	1953 ~1957	(Xu, J. *et al.* 2015)[89]

Asia	China, Xinjiang, Kuytun	Present	1953 ~1957	(Xu, J. *et al.* 2015)[89]
Asia	China, Xinjiang, Nilka	Present	1953 ~1957	(Xu, J. *et al.* 2015)[89]
Asia	China, Xinjiang, Shawan	Present	1953 ~1957	(Xu, J. *et al.* 2015)[89]
Asia	China, Xinjiang, Tekes	Present	1953 ~1957	(Xu, J. *et al.* 2015)[89]
Asia	China, Xinjiang, Wusu (Usu)	Present	1953 ~1957	(Xu, J. *et al.* 2015)[89]
Asia	China, Xinjiang, Xinyuan	Present	1953 ~1957	(Xu, J. *et al.* 2015)[89]
Asia	China, Xinjiang, Yumin	Present	1953 ~1957	(Xu, J. *et al.* 2015)[89]
Oceania	Australia, Australian Capital Territory	Present	1957	(Geier, P. W. 1963)[107]
Asia	Russian, Khabarovsk	Present	1958	(Wearing, C. H. *et al.* 2001)[108]
Europe	Slovakia	Present	1961	(Vavrovič, J. *et al.* 2014)[109]
Europe	Hungary	Present	1963	(Bodor, J. 1969)[110]
North America	Mexico	Present	1963	(Wearing, C. H. *et al.* 2001)[108]
Asia	India, Himachal Pradesh	Present	1964	(Khajuria, D. R. *et al.* 1986)[90]
Europe	France, Northern France	Widespread	1928	(Rosenberg, H. T. 1934)[111]
Africa	Egypt, Alexandria	Present	1965	(ElGamil, F. M. *et al.* 1977)[112]
Asia	Russia, Southern Primor'ye	Present	1967	(Shel'deshova, G. G. 1967)
Asia	Russia, Vladivostok	Present	1967	(Shel'deshova, G. G. 1967)
Europe	Lithuania	Present	1969	EPPO 2014
Africa	Tunisia	Present	1976	EPPO 2014
Europe	Czechoslovakia (former)	Present	1980	(Komarek, S. 1987)[113]
Europe	Poland, Wielkopolsk	Present	1981	(Kozłowski, J. 1994)[114]
Europe	South Sweden	Present	1981	(Subinprasert, S. 2010)[115]
Asia	Azerbaijan	Present	1983	(Zhigarevich, G. P. & Yakubov, Z. B. 1990)[116]
Asia	Iraq, Tarmiya and Madain	Present	1983	(Ahmad, T. R. 1988)[117]
Europe	Romania	Present	1983	(**Minoiu, N.** & **Boaru, M.** 1989)[118]
Asia	Israel	Present	1984	(Steinberg, S. *et al.* 1988)[119]
Asia	Turkey	Present	1984	Bahriye H. *et al.* 1984
Asia	India, Jammu and Kashmir	Present	1985	(Pawar, A. D. & Tuhan, N. C. 1985)[120]
Asia	India, Himachal Pradesh	Present	Before 1986	(Khajuria, D. R. *et al.* 1986)[90]
Asia	Kinnaur district	Present	Before 1986	(Khajuria, D. R. *et al.* 1986)[90]
Europe	Ukraine	Present	1986	(Stefanovska, T. R. *et al.* 2000)[121]
Europe	Swiss	Present	1986	(Minks, A. K. 1997)[122]
Asia	China, Xinjiang, Tumushuke (Toumchouq)	Present	Before 1987	(Xu, J. *et al.* 2015)[89]
Asia	China, Xinjiang, Artux	Present	Before 1987	(Xu, J. *et al.* 2015)[89]
Asia	China, Xinjiang, Awant	Present	Before 1987	(Xu, J. *et al.* 2015)[89]
Asia	China, Xinjiang, Bole	Present	Before 1987	(Xu, J. *et al.* 2015)[89]
Asia	China, Xinjiang, Changji	Present	Before 1987	(Xu, J. *et al.* 2015)[89]
Asia	China, Xinjiang, Fukang	Present	Before 1987	(Xu, J. *et al.* 2015)[89]
Asia	China, Xinjiang, Hami	Present	Before 1987	(Xu, J. *et al.* 2015)[89]
Asia	China, Xinjiang, Hoxu	Present	Before 1987	(Xu, J. *et al.* 2015)[89]
Asia	China, Xinjiang, Akqi	Present	Before 1987	(Xu, J. *et al.* 2015)[89]
Asia	China, Xinjiang, Akto	Present	Before 1987	(Xu, J. *et al.* 2015)[89]
Asia	China, Xinjiang, Bachu	Present	Before 1987	(Xu, J. *et al.* 2015)[89]
Asia	China, Xinjiang, Barkol	Present	Before 1987	(Xu, J. *et al.* 2015)[89]
Asia	China, Xinjiang, Bohu	Present	Before 1987	(Xu, J. *et al.* 2015)[89]
Asia	China, Xinjiang, Burqin	Present	Before 1987	(Xu, J. *et al.* 2015)[89]
Asia	China, Xinjiang, Fuhai	Present	Before 1987	(Xu, J. *et al.* 2015)[89]
Asia	China, Xinjiang, Fuyun	Present	Before 1987	(Xu, J. *et al.* 2015)[89]
Asia	China, Xinjiang, Habahe	Present	Before 1987	(Xu, J. *et al.* 2015)[89]

Asia	China, Xinjiang, Hejing	Present	Before 1987	(Xu, J. *et al.* 2015)[89]
Asia	China, Xinjiang, Hutubi	Present	Before 1987	(Xu, J. *et al.* 2015)[89]
Asia	China, Xinjiang, Jeminay	Present	Before 1987	(Xu, J. *et al.* 2015)[89]
Asia	China, Xinjiang, Jimsar	Present	Before 1987	(Xu, J. *et al.* 2015)[89]
Asia	China, Xinjiang, Kalpin	Present	Before 1987	(Xu, J. *et al.* 2015)[89]
Asia	China, Xinjiang, Lop	Present	Before 1987	(Xu, J. *et al.* 2015)[89]
Asia	China, Xinjiang, Markit	Present	Before 1987	(Xu, J. *et al.* 2015)[89]
Asia	China, Xinjiang, Minfeng	Present	Before 1987	(Xu, J. *et al.* 2015)[89]
Asia	China, Xinjiang, Mori	Present	Before 1987	(Xu, J. *et al.* 2015)[89]
Asia	China, Xinjiang, Pishan	Present	Before 1987	(Xu, J. *et al.* 2015)[89]
Asia	China, Xinjiang, Qapqal	Present	Before 1987	(Xu, J. *et al.* 2015)[89]
Asia	China, Xinjiang, Qiemo	Present	Before 1987	(Xu, J. *et al.* 2015)[89]
Asia	China, Xinjiang, Qinghe	Present	Before 1987	(Xu, J. *et al.* 2015)[89]
Asia	China, Xinjiang, Qira	Present	Before 1987	(Xu, J. *et al.* 2015)[89]
Asia	China, Xinjiang, Qitai	Present	Before 1987	(Xu, J. *et al.* 2015)[89]
Asia	China, Xinjiang, Ruoqiang	Present	Before 1987	(Xu, J. *et al.* 2015)[89]
Asia	China, Xinjiang, Shufu	Present	Before 1987	(Xu, J. *et al.* 2015)[89]
Asia	China, Xinjiang, Shule	Present	Before 1987	(Xu, J. *et al.* 2015)[89]
Asia	China, Xinjiang, Toli	Present	Before 1987	(Xu, J. *et al.* 2015)[89]
Asia	China, Xinjiang, Toksun	Present	Before 1987	(Zhao, L. *et al.* 2015)[123]
Asia	China, Xinjiang, Wuqia	Present	Before 1987	(Xu, J. *et al.* 2015)[89]
Asia	China, Xinjiang, Wushi	Present	Before 1987	(Xu, J. *et al.* 2015)[89]
Asia	China, Xinjiang, Yanqi	Present	Before 1987	(Xu, J. *et al.* 2015)[89]
Asia	China, Xinjiang, Yecheng	Present	Before 1987	(Xu, J. *et al.* 2015)[89]
Asia	China, Xinjiang, Yengisar	Present	Before 1987	(Xu, J. *et al.* 2015)[89]
Asia	China, Xinjiang, Yiwu	Present	Before 1987	(Xu, J. *et al.* 2015)[89]
Asia	China, Xinjiang, Yuepuhu (Yopurga)	Present	Before 1987	(Xu, J. *et al.* 2015)[89]
Asia	China, Xinjiang, Yuli	Present	Before 1987	(Xu, J. *et al.* 2015)[89]
Asia	China, Xinjiang, Yutian	Present	Before 1987	(Xu, J. *et al.* 2015)[89]
Asia	China, Xinjiang, Zepu	Present	Before 1987	(Xu, J. *et al.* 2015)[89]
Asia	China, Xinjiang, Zhaosun	Present	Before 1987	(Xu, J. *et al.* 2015)[89]
Asia	North Korea	Present	1987	(Xu, J. *et al.* 2015)[89]
Asia	China, Gansu, Dunhuang	Present	1987	(Qin, X. H. *et al.*2006)[124]
Africa	Algeria	Present	1989	EPPO
Africa	Libya	Present	1989	EPPO
Pasia	Jordan	Present	1989	EPPO
Asia	Kyrgyzstan	Present	1989	EPPO
Asia	Lebanon	Present	1989	EPPO
Asia	Tajikistan	Present	1989	EPPO
Asia	Turkmenistan	Present	1989	EPPO
Asia	Uzbekistan	Present	1989	EPPO
Asia	Cyprus	Present	1989	EPPO
Europe	Belarus	Widespread	1989	EPPO
Europe	Denmark	Present	1989	EPPO
Europe	Latvia	Present	1989	EPPO
Europe	Moldova	Present	1989	EPPO
Europe	Norway	Present	1989	EPPO
Oceania	South Australia	Present	1989	EPPO
South America	Chile	Present	1989	EPPO
South America	Peru	Present	1992	CABI, 2002&2007
South America	Brazil, Vacaria	Restricted distribution	1991	(Kovaleski & Mumford, 2007)[125]
Asia	China, Gansu, Jiuquan	Present	1992	(Qin, X. H. *et al.* 2006)[124]
Europe	Spain, Lleida	Present	1993	(Giner, M. 2014)[126]

Asia	China, Gansu, Guazhou	Present	1994	(Zhao, L. *et al.* 2015)[123]
Asia	China, Gansu, Yumen	Present	1994	(Zhao, L. *et al.* 2015)[123]
Asia	China, Gansu, Qinghai	Present	1994	(Wearing, C. H. *et al.* 2001)[108]
Asia	China, Ningxia	Present	1994	(Wearing, C. H. *et al.* 2001)[108]
Asia	China, Shaanxi	Present	1994	(Wearing, C. H. *et al.* 2001)[108]
Asia	China, Shanxi	Present	1994	(Wearing, C. H. *et al.* 2001)[108]
Asia	Pakistan, Quetta valley	Present	1994	(Asmatullah-Kakar & Hazara, A. H. 2009)[127]
Asia	Gansu, Suzhou	Present	1995	(Xu, J. *et al.* 2015)[89]
Europe	Ireland	Present	1995	EPPO
Europe	Malta	Present	1995	EPPO
Europe	Albania	Present	1996	EPPO
Europe	Estonia	Present	1996	EPPO
Asia	China, Gansu, Jinta	Present	1999	(Xu, J. *et al.* 2015)[89]
Asia	China, Gansu, Anxi	Present	2000	(Xu, J. *et al.* 2015)[89]
Europe	Austria	Present	Before 2000	(Polesny, F. 2000)[128]
Europe	southern Scandinavia	Present	Before2001	(Wearing, C. H. *et al.* 2001)[108]
Europe	Portuguesa, Madeira	Present	Before 2001	(Wearing, C. H. *et al.* 2001)[108]
Europe	Russia, Eastern Siberia	Present	Before 2001	(Wearing, C. H. *et al.* 2001)[108]
Africa	Spain, Canary Islands	Present	Before 2001	(Wearing, C. H. *et al.* 2001)[108]
Asia	China, Gansu, Gaotai	Present	2003	(Xu, J. *et al.* 2015)[89]
Asia	China, Gansu, Linze	Present	2004	(Wang, L. *et al.* 2011)[104]
Asia	China, Gansu, Ganzhou	Present	2005	(Xu, J. *et al.* 2015)[89]
Asia	China, Gansu, Minle	Present	2005	(Xu, J. *et al.* 2015)[89]
Asia	China, Gansu, Zhangye	Present	2005	(Qin, X. H. *et al.* 2006)[124]
Asia	China, Gansu, Sunan	Present	2006	(Xu, J. *et al.* 2015)[89]
Asia	China, Gansu, Shandan	Present	2006	(Wang, L. *et al.* 2011)[104]
Asia	China, Gansu, Jiayuguan	Present	2006	(Xu, J. *et al.* 2015)[89]
Asia	China, Heilongjiang	Present	2006	(Liu, Y. Y. *et al.* 2012)[129]
Asia	China, Heilongjiang, Dongning	Present	2006	(Xu, J. *et al.* 2015)[89]
Asia	China, Nei Menggu (Inner Mongolia Autonomous Region)	Present	2006	(Xu, J. *et al.* 2015)[89]
Asia	China, Neimeng, Ejinaqi (Ejin / Ejin Qi)	Present	2006	(Liu, Y. Y. *et al.* 2012)[129]
Asia	China, Gansu, Lanzhou	Present	2007	(Zhang, R. Z. *et al.* 2012)[130]
Asia	China, Gansu, Qilihe	Present	2007	(Xu, J. *et al.* 2015)[89]
Asia	China, Gansu, Xigu	Present	2007	(Xu, J. *et al.* 2015)[89]
Asia	China, Gansu, Yongchang	Present	2007	(Xu, J. *et al.* 2015)[89]
Asia	China, Gansu, Minqin	Present	2007	(Xu, J. *et al.* 2015)[89]
Asia	China, Heilongjiang, Hailin	Present	2007	(Xu, J. *et al.* 2015)[89]
Asia	China, Heilongjiang, Hulin	Present	2007	(Xu, J. *et al.* 2015)[89]
Asia	China, Heilongjiang, Jidong	Present	2007	(Xu, J. *et al.* 2015)[89]
Asia	China, Heilongjiang, Jixi, Jiguan	Present	2007	(Xu, J. *et al.* 2015)[89]
Asia	China, Heilongjiang, Jixi, Hengshan	Present	2007	(Xu, J. *et al.* 2015)[89]
Asia	China, Heilongjiang, Jixi, Chengzihe	Present	2007	(Xu, J. *et al.* 2015)[89]
Asia	China, Heilongjiang, Linkou	Present	2007	(Xu, J. *et al.* 2015)[89]
Asia	China, Heilongjiang, Mishan	Present	2007	(Xu, J. *et al.* 2015)[89]
Asia	China, Heilongjiang, Mudanjiang, Dong'an	Present	2007	(Xu, J. *et al.* 2015)[89]
Asia	China, Heilongjiang, Mudanjiang, Yangming	Present	2007	(Xu, J. *et al.* 2015)[89]
Asia	China, Heilongjiang, Mudanjiang, Aimin	Present	2007	(Xu, J. *et al.* 2015)[89]

Asia	China, Heilongjiang, Mudanjiang, Xi'an	Present	2007	(Xu, J. *et al.* 2015)[89]
Asia	China, Heilongjiang, Ning'an	Present	2007	(Xu, J. *et al.* 2015)[89]
South America	Brzail, Caxias do Sul	Present	2007	(Michael, J. W. *et al.* 2009)[131]
South America	Brazil, Rio Grande do Sul, Vacaria	Restricted distribution	2007	(Michael, J. W. *et al.* 2009)[131]
Asia	China, China, Gansu, Chengguan	Present	2008	(Xu, J. *et al.* 2015)[89]
Asia	China, Gansu, Ning'an	Present	2008	(Xu, J. *et al.* 2015)[89]
Asia	China, Gansu, Jingtai	Present	2008	(Zhao, L. *et al.* 2015)[123]
Asia	China, Gansu, Gaolan	Present	2008	(Xu, J. *et al.* 2015)[89]
Asia	China, Gansu, Liangzhou	Present	2008	(Zhao, L. *et al.* 2015)[123]
Asia	China, Gansu, Baiyin	Present	2008	(Xu, J. *et al.* 2015)[89]
Asia	China, Gansu, Pingliang	Present	2008	---
Asia	China, Ningxia, Zhongwei, Zhongning, Xinbao	Present	2008	(Wang, H. M. 2014)[132]
Asia	China, Ningxia, Zhongwei, Shapotou	Present	2008	(Xu, J. *et al.* 2015)[89]
Asia	China, Ningxia, Zhongning,	Present	2008	(Xu, J. *et al.* 2015)[89]
Asia	China, Neimeng, Alashanzuoqi (Alxa LB/Alxa Zuoqi)	Present	2008	(Zhang, R. Z. *et al.* 2012)[130]
South America	Brazil, Parana	Present	Before 2009	(Michael, J. W. *et al.* 2009)[131]
South America	Brazil, Santa Catarina, Lages	Present	Before 2009	(Michael, J. W. *et al.* 2009)[131]
Asia	China, Ningxia, Qingtongxia	Present	2009	(Xu, J. *et al.* 2015)[89]
Asia	China, Gansu, Gulang	Present	2009	(Xu, J. *et al.* 2015)[89]
Asia	China, Gansu, Yongdeng	Present	2009	(Xu, J. *et al.* 2015)[89]
Asia	China, Neimeng, Wuhai, Haibowan	Present	2009	(Xu, J. *et al.* 2015)[89]
Asia	China, Jilin, Hunchun	Present	2009	(Xu, J. *et al.* 2015)[89]
Asia	China, Neimeng, Alashanyouqi (Alxa RB/Alxa Youqi	Present	2010	(Xu, J. *et al.* 2015)[89]
Asia	China, Gansu, Jinchuan	Present	2010	(Xu, J. *et al.* 2015)[89]
Asia	China, Gansu, Jingyuan	Present	2012	(Xu, J. *et al.* 2015)[89]
Asia	China, Gansu, Honggu	Present	2012	(Xu, J. *et al.* 2015)[89]
Asia	China, Heilongjiang, Jixian	Present	2012	(Xu, J. *et al.* 2015)[89]
Asia	China, Heilongjiang, Baoqing	Present	2012	(Xu, J. *et al.* 2015)[89]
Asia	China, Heilongjiang, Boli	Present	2012	(Xu, J. *et al.* 2015)[89]
Asia	China, Heilongjiang, Jiamusi	Present	2012	(Xu, J. *et al.* 2015)[89]
Asia	China, Heilongjiang, Zhaozhou	Present	2013	(Xu, J. *et al.* 2015)[89]
Asia	China, Heilongjiang, Qitaihe, Xinxing	Present	2013	(Xu, J. *et al.* 2015)[89]
Asia	China, Heilongjiang, Qitaihe, Taoshan	Present	2013	(Xu, J. *et al.* 2015)[89]
Asia	China, Liaoning, Haicheng	Present	2012	(Xu, J. *et al.* 2015)[89]
Asia	China, Liaoning, Suizhong	Present	2013	(Xu, J. *et al.* 2015)[89]
Asia	China, Liaoning, Jianchang	Present	2013	(Xu, J. *et al.* 2015)[89]
Asia	India, Uttar Pradesh	Present	---	---
Asia	China, Shandong	Present	---	(Xu, J. *et al.* 2015)[89]
Europe	Czech Republic	Present	---	(Zichová, T. *et al.* 2011)[133]
Europe	France, Corsica	Present	---	---
Europe	Italy, Sardinia	Present	---	---
Europe	Italy, Sicily	Present	---	---
Europe	Portuguesa, Azores	Present	---	---
Europe	Russia, Western Siberia	Present	---	---
Europe	Yugoslavia (Serbia and Montenegro)	Present	---	---
North America	USA, Minnesota	Present	---	(Cook, W.C. 1921)[134]
North America	USA, Missouri	Present	---	(Haseman, L. 1934)[135]

North America	USA, North Carolina	Present	---	---
North America	USA, Virginia	Present	---	---
North America	USA, Wisconsin	Present	---	---
Oceania	Australia, Queensland	Present	---	---
Oceania	Australia, Victoria	Present	---	---

Supplementary Table 2. Statistics of genomic sequencing data of *Cydia pomonella* by Illumina HiSeq 2000

Libraray	Size (bp)	Read length (bp)	Raw-data	Clean-data (Gb)	Estimated coverage (x)
Paired-end	180	2*101	47,744,883,216	47,744,883,216	76
Paired-end	300	2*101	45,236,112,502	45,236,112,502	72
Paired-end	500	2*101	42,492,543,250	42,492,543,250	67
Paired-end	800	2*101	36,305,891,068	40,903,439,954	65
Mate-pair	3Kb	2*101	44,392,313,456	22,503,215,966	36
Mate-pair	8Kb	2*101	41,768,966,322	23,143,629,913	37
Mate-pair	10Kb	2*101	41,528,214,642	23,478,350,796	37
	**Total**		299,468,924,456	245,502,175,597	390

*Assumed genome size to be 630Mb

Supplementary Table 3. Estimation of *Cydia pomonella* genome size by K-mer analysis

K-mer individual sum	K-mer depth coverage	Estimate genome size(bp)	Heterozygosity
134,256,472,034	212	633,285,245	0.6%

Supplementary Table 4. Statistics of genomic sequencing data of *Cydia pomonella* by Pacbio RS II

Cell ID	Polymerase Read Bases (bp)	Polymerase Reads
1	1,187,579,745	90,781
2	1,217,858,553	91,414
3	1,014,491,166	77,137
4	1,129,985,366	85,466
5	U 54,763,783	89,442
6	1.236,739,658	93.726
7	1,216,663,308	92,172
8	1,040,376,198	79,506
9	1,096,533,930	87,208
10	1,01 1,837,100	81,555
11	1,072,503,510	84,177
12	1,125,429,218	88,038
13	1,176,346,335	88,034
14	1,162,684,775	87,425
15	751,480.28	61.587
16	1,033,090,752	85,797
17	1,092,401,792	86,160
18	1J 13,311,294	87,923
19	1,168 J 50.779	92,694
20	1,220,762,179	95,825
21	1,163,151,280	93,902
22	1,104,931,542	88,175
23	663,105,476	45,441
24	1,152,935,712	91,107
25	1,001,677,141	82,270
26	1,010,128,388	81,664
27	1,206,327.46	93,998
28	881,247,819	65,032
29	780,353,987	57,952
30	688,020,137	50,700
31	519,305,950	42,546
32	682,653,090	51,513
33	5,754,489,744	539,512
34	6,910,992,144	657,757
35	5,621,702,410	534,770
36	4,738,995,638	451,770

37	5,144,507,815	472,299
38	968,699,284	351,533
**Total**	54,575,562,192	5,422,850

Supplementary Table 5. Statistics of genomic sequencing data of *Cydia pomonella* by BioNano

Enzyme	BssSI	BspQI
Quantity (Gb)	552.772	419.5318
Avg. N50 (Mb) (>=150Kb)	0.2064	0.395
Avg. N50 (Mb) (>=20Kb)	0.11	0.3265
Avg. Label Density (per 100 Kb)	14.37	6.51
Avg. Map Rate (%)	22.2	8.4
Estimated Effective Coverage	202.0×	58.6×
Avg. False Positive	12.0% 2.16/100kbp	22.3% 1.16/100kbp
Avg. False Negative	15.40%	8.50%
Scans Completed	55	62

Supplementary Table 6. Statistics of genomic sequencing data of *Cydia pomonella* by Hi-C

Read length (bp)	Sequencer	Read length (bp)	Raw-data (bp)	Clean-data (bp)
2x100	Illumina 4000	2*100	95,576,313,600	88,158,552,200

Supplementary Table 7. Summary of *Cydia pomonella* genome assembly

Category	Contigs	Scaffolds	Chromosomes
Total length (bp)	682,491,354	772,891,954	772,999,854
Max length (bp)	5,711,842	34,601,981	58,169,538
Average length (bp)	307,290	450,140	1,211,598
N20 length	1,849,489	19,535,149	38,641,383
N50 length (bp)	862,490	8,915,549	28,370,328
N90 length (bp)	118,606	130,046	14,500,452
Total sequence numbers	2,221	1,717	638

Supplementary Table 8. The chromosome statistics of different Lepidoptera species

Species	Number of assembled chromosomes	Coverage (%)[1]	BUSCO3 Assessment
Cydia pomonella	29	97.48	C[2]:97.8% [S[3]:94%, D[4]:3.8%], F[5]:0.7%, M[6]:1.5%
Trichoplusia ni	28	90.62	C:97.4% [S:92.3%, D:5.1%], F:0.4%, M:2.2%
Spodoptera litura	31	91.09	C:97.2% [S:95.8%, D:1.4%], F:1.1%, M:1.7%
Bombyx mori	28	87.30	C:95.8% [S:95.4%, D:0.4%], F:1.9%, M:2.3%
Melitaea cinxia	31	72.45	C:82.9% [S:82.8%, D:0.1%], F:8.6%, M:8.5%
Heliconius melpomene	21	82.68	C:95.6% [S:95.1%, D:0.5%], F:1.8%, M:2.6%

[1]The percentage of all chromosome's length in whole genome size.

[2]C: The percentage of complete length of 1,658 insect conserved genes

[3]S: The percentage of complete length but not duplication of 1,658 insect conserved genes

[4]D: The percentage of complete length but duplication of 1,658 insect conserved genes

[5]F: The percentage of fragment length of 1,658 insect conserved genes

[6]M: The percentage of missing finding of 1,658 insect conserved genes

Supplementary Table 9. The sequencing data statistic of Nanopore sequencing of *Cydia pomonella*

Feature	Number
of_total_Reads_Bases (bps)	83,473,696,842
of_total_Reads_Number	4,161,465
Pass_Reads_Bases (bps)	71,105,727,881
Pass_Reads_Number	3,068,220
Pass_Reads_Mean_Length (bps)	23174.91
Pass_Reads_N50_Length (bps)	32,637
Pass_Reads_Medium_Length (bps)	18,322
Pass_Reads_Max_Length (bps)	223,241

Supplementary Table 10. The statistics of the full-length transcripts mapped to reference genome of *Cydia pomonella*

Feature	Number
Total Reads	25,940
Mapped Reads	24,326
Mapping Rate	0.937779
UnMapped Reads	1,614
MultiMap Reads	782
MultiMap Rate	0.030146
Reads Mapping Forward	11,822
Reads Mapping Reverse	11,722

Supplementary Table 11. Classification of repeat sequences identified in the *Cydia pomonella* genome

Repeat types		Number of elements*	Length occupied (bp)	Percentages of sequence (%)
Interspersed repeats	SINE	116,758	21,027,453	2.72
	LINE	284,745	68,510,592	8.86
	LTR	21,431	11,392,329	1.47
	DNA elements	113,169	26,949,964	3.49
	Unclassified	979,634	197,774,975	25.59
Small RNA		57,136	10,389,673	1.34
Satellites		3,534	419,534	0.05
Simple repeats		115,808	5,078,280	0.66
Total base masked		1,692,215	341,542,800	42.87

Supplementary Table 12. Statistics of RNA-Seq data of *Cydia pomonella*

Sample	Experiment Title	Instrument	Layout	Total Bases	SRA Accession
adult	Female adult after hot treatment	Illumina	PAIRED	5,028,864,336	-
egg	Egg of 1 day	Illumina	PAIRED	6,403,958,820	-
egg	Egg of 4 day	Illumina	PAIRED	5,861,513,196	-
egg	Mixed eggs	Illumina	PAIRED	6,336,913,968	-
larva	5-star larva	Illumina	PAIRED	5,967,277,092	-
larva	Larva of female	Illumina	PAIRED	5,369,661,364	-
larva	Larva of male	Illumina	PAIRED	4,168,167,788	-
pupa	Pupa of female	Illumina	PAIRED	5,755,115,946	-
abdomen	Abdomen of female adult	Illumina	PAIRED	4,699,588,580	-
abdomen	Abdomen of male adult	Illumina	PAIRED	4,990,923,888	-
antennae	*Cydia pomonella* Adult Female Antennae	Illumina	PAIRED	10,940,081,236	SRX1082030
antennae	*Cydia pomonella* Adult Male Antennae	Illumina	PAIRED	12,774,512,522	SRX1082029
accessory gland	Accessory gland 1	Illumina	PAIRED	4,319,808,642	SRX2068935
accessory gland	Accessory gland 2	Illumina	PAIRED	4,680,433,471	SRX2068936
head	*Cydia pomonella* Neonate Larval Heads	Illumina	PAIRED	9,755,633,026	SRX1082032
head	Head female	Illumina	PAIRED	5,298,647,067	SRX2068932
head	Head male	Illumina	PAIRED	3,729,928,790	SRX2068938
midgut	Midgut female rep 1	Illumina	PAIRED	3,710,357,801	SRX2068939
midgut	Midgut female rep 2	Illumina	PAIRED	3,541,381,034	SRX2068940
midgut	Midgut male rep 1	Illumina	PAIRED	4,634,689,190	SRX2068941
midgut	Midgut male rep 2	Illumina	PAIRED	4,258,648,766	SRX2068942
ovary	Ovary 1	Illumina	PAIRED	4,808,921,346	SRX2068943
ovary	Ovary 2	Illumina	PAIRED	6,077,116,796	SRX2068944
testis	Testis 1	Illumina	PAIRED	4,800,261,231	SRX2068933
testis	Testis 2	Illumina	PAIRED	6,108,103,903	SRX2068934
	**Total Bases (bp)**			**166,073,078,192**	

Supplementary Table 13. Predicted corresponding *CpomOR* names of annotated OGS sequences

GeneID	OrID	GeneID	OrID	GeneID	OrID
CPOM22313	OR10a	CPOM22373	OR26a	CPOM22309	OR42a
CPOM22301	OR10b	CPOM22352	OR27a	CPOM22310	OR43a
CPOM22358	OR11a	CPOM22353	OR28a	CPOM22378	OR43b
CPOM22359	OR11b	CPOM22377	OR28b	CPOM22307	OR44a
CPOM22360	OR11c	CPOM22332	OR29a	CPOM22367	OR44b
CPOM22371	OR11d	CPOM22333	OR29b	CPOM22324	OR46a
CPOM22342	OR12a	CPOM22302	OR3b	CPOM22325	OR47a
CPOM22314	OR12b	CPOM22303	OR3a	CPOM22305	OR5a
CPOM22297	OR12c	CPOM22370	OR33a	CPOM22348	OR53a
CPOM22311	OR12d	CPOM22322	OR30a	CPOM22380	OR54a
CPOM22375	OR14a	CPOM22323	OR30b	CPOM22318	OR56a
CPOM22347	OR15a	CPOM22334	OR31a	CPOM22319	OR56b
CPOM22337	OR16a	CPOM22335	OR31b	CPOM22320	OR56c
CPOM22312	OR16b	CPOM22351	OR32a	CPOM22304	OR58a
CPOM22354	OR18a	CPOM22330	OR35a	CPOM22339	OR6a
CPOM22306	OR19a	CPOM22331	OR35b	CPOM22340	OR6b
CPOM22296	OR19b	CPOM22355	OR36a	CPOM22341	OR6c
CPOM22344	Orco	CPOM22356	OR36b	CPOM22368	OR6d
CPOM22361	OR2a	CPOM22315	OR38a	CPOM22369	OR6e
CPOM22362	OR2b	CPOM22316	OR38b	CPOM22343	OR60a
CPOM22363	OR2c	CPOM22317	OR38c	CPOM22379	OR61a
CPOM22365	OR2d	CPOM22321	OR38d	CPOM22346	OR61b
CPOM22374	OR20a	CPOM22345	OR39a	CPOM22328	OR63a
CPOM22357	OR21a	CPOM22338	OR4a	CPOM22300	OR67a
CPOM22376	OR22a	CPOM22372	OR4b	CPOM22299	OR68a
CPOM22329	OR22b	CPOM22364	OR4c	CPOM22350	OR72a
CPOM22326	OR24a	CPOM22366	OR4d	CPOM22349	OR8a
CPOM22327	OR24b	CPOM22336	OR40a	CPOM22308	OR85a

Supplementary Table 14. Summary of the different gene family with the insecticide resistance

Gene families	Sub families	Numbers of genes	Total genes
P450	Clan2	8	136
	Clan3	67	
	Clan4	47	
	Mito	14	
CCE	ie	3	73
	glu	1	
	be	4	
	gli	2	
	jhe	1	
	nlg	7	
	ae	20	
	lepdopteran esterases	35	
GST	Delta	6	30
	Epsilon	11	
	Omega	2	
	GDAP1	4	
	Zeta	1	
	Theta	1	
	Sigma	2	
	AIMP3	2	
	others	1	
ABC	ABCC	16	47
	ABCD	3	
	ABCE	0	
	ABCF	2	
	ABCB	12	
	ABCH	2	
	ABCA	4	
	ABCG	8	
nAChR		9	9
ACE	ACHE1	1	2
	ACHE2	1	
VGSC		1	1

Supplementary Table 15. Gene features of *Cydia pomonella*, *Bombyx mori* and *Danaus plexippus*

Species	Gene Length	Exon Number	Exon Length	Intron Length	CDS Length
C. pomonella	6033.60	5.68	256.91	1205.29	1460.95
B. mori	6028.52	5.44	223.81	1288.74	1218.74
D. plexippus	6001.37	6.71	204.97	996.50	1376.10

Supplementary Table 16. Small RNA sequencing of *Cydia pomonella*

Sample	Total Bases	Read Count	low_quality	3'adapter_null	insert_null	5'adapter_contaminants	smaller_than_18nt	clean_reads
Small RNA	1,579,068,273	30,962,123	596,145(1.93%)	44,048(0.14%)	11,032(0.04%)	52,979(0.17%)	618,859(2.00%)	29,638,344(95.72%)

Supplementary Table 17. Noncoding RNA of *Cydia pomonella*

**Type**	***Cydia pomonella***
ribosomal RNA (rRNA)	334
microRNA(miRNA)	217
Piwi-interacting RNA (piRNA)	137,751
transfer RNA (tRNA)	2,435
snoRNA	82

Supplementary Table 18. The statistics on different type of orthologous gene groups

Species	1:1:1	N:N:N	Diptera	Coleoptera	Hymenoptera	Hemiptera	Isoptera	Lepidoptera	Moth	Butterfly	Others	SD	ND
Locusta migratoria	2124	2051	0	0	0	0	0	0	0	0	2618	544	14042
Zootermopsis nevadensis	2014	1888	0	0	0	0	940	0	0	0	3351	133	6284
Cryptotermes secundus	2051	2011	0	0	0	0	940	0	0	0	3433	226	9393
Bemisia tabaci	2028	1969	0	0	0	50	0	0	0	0	2898	493	8224
Rhodnius prolixus	1960	1874	0	0	0	50	0	0	0	0	2815	415	7950
Apis mellifera	2101	2032	0	0	417	0	0	0	0	0	3216	132	7416
Nasonia vitripennis	1992	1948	0	0	417	0	0	0	0	0	2832	673	10869
Anoplophora glabripennis	2106	2023	0	544	0	0	0	0	0	0	3477	254	6424
Tribolium castaneum	2087	2029	0	544	0	0	0	0	0	0	3381	288	8197
Anopheles gambiae	2071	2010	176	0	0	0	0	0	0	0	2762	332	5670
Drosophila melanogaster	2076	1996	176	0	0	0	0	0	0	0	2702	416	6553
Plutella xylostella	1815	1921	0	0	0	0	0	616	55	0	2777	370	10519
Cydia pomonella	2029	1977	0	0	0	0	0	508	48	0	2956	574	8406
Spodoptera litura	2119	2044	0	0	0	0	0	700	90	0	3421	209	7599
Trichoplusia ni	2018	1974	0	0	0	0	0	649	72	0	2814	112	6398
Bombyx mori	2058	2014	0	0	0	0	0	659	70	0	3181	100	6541
Manduca sexta	2089	2035	0	0	0	0	0	686	87	0	3243	150	7161
Melitaea cinxia	2124	2051	0	0	0	0	0	702	0	230	3141	59	8360
Heliconius melpomene	2124	2051	0	0	0	0	0	702	0	193	3044	43	4512
Danaus plexippus	2124	2051	0	0	0	0	0	702	0	194	3493	72	6494

Supplementary Table 19. Classification and annotation of the four W-enriched repeats of *Cydia pomonella*

Repeat	Estimated female genome proportion ± S.D.	Presence on chr.29	Class	Subclass/ order	Superfamily	Monomer length	Annotation of consensus sequence	GenBank Acc.No.
A	0.07 ± 0.017 %	Partial sequence	retro-transposon	LTR	Bel-Pao	6.4 kbp	516 bp long terminal repeat on both ends; 3675-4331 RT domain	MK626522
B	0.068 ± 0.009 %	YES	satellite			172 bp		MK626521
C	0.035 ± 0.024 %	NO	DNA transposon	TIR	hAT	2.7 kbp	63-1649 Transposase	MK626520
D	0.023 ± 0.003 %	Partial sequence	retro-transposon	LINE		1.8 kbp	227-802 RNA dependent DNA polymerase	MK626519

Notes to W chromosome (chr. 29) in assembly V6:

1) **Not all predicted W enriched repeats are present on Chr. 29**
2) ***Cydia* W-specific sequences CpW2** (acc. no. AM292090) **and CpW5** (acc. no. AM292091) (see Fuková et al. 2007) **are both present on Chr. 14.**

Supplementary Table 20. Primers used for probe synthesis in two-color FISH test of *Cydia pomonella*. Either Digoxigenin (Dig)- or Biotin (Bio)-labeled probes were synthesized according to pairs of tested genes. Treatments included *CpomOrco/OR3a*, *CpomOrco/OR3b*, and *CpomOR3a/3b*, respectively. In order to better identify co-localizations of genes, Dig signals were adjusted to green color and Bio signals were adjusted to purple.

Gene	Primer	Label
CpomOrco	5'-CGAACTCACCGCCAATACCATCACGGTCTTGTTCTTTGC-3'	Digoxigenin
	3'-GACACCAACATGTGAAATAGTAGAGCAGTACC-5'	
CpomOR3a	5'-CTAACAAGATTTATAAAAAAATAG-3'	Digoxigenin,
	5'-AGGTCACCGTACGAAGCATAATAAAATATGAAC-3'	Biotin
CpomOR3b	5'-CAAACAAGATTTATAAGAACGTCG-3'	Biotin
	5'-TAGTCACAGTGCGAAGCATAATATAATATGAAA-3'	

Supplementary Table 21. Sequence information of siRNA primers. siRNAs were chemically synthesized by Shanghai Gene Pharma with 2' Fluoro dU modification to increase the stability. siGFPs were designed in order to assess possible off-target effects. For insecticide resistance tests, 0.5 µl siRNA was injected into larva; for chemosensory tests, 1 µl siRNA was injected into pupa.

Gene name	Primer	siRNA sequence (5'-3')
GFP	F	UGCGCUCCUGGACGUAGCCTT
	R	CTACGUCCAGGAGCGCACCTT
CYP6B2	F	GGAAGUCAAGAGGGCUCAUTT
	R	AUGAGCCCUCUUGACUUCCTT
OR3a & OR3b	F	CCCUAAACCUGCUAAUCAUTT
	R	AUGAUUAGCAGGUUUAGGGTT
OR3a	F	UAUUUUUUUAUAAAUCUUGUU
	R	CAAGAUUUAUAAAAAAAUAGA
OR3b	F	UAGAGAUUCGGAGUUCAAGGU
	R	CUUGAACUCCGAAUCUCUAGG

Supplementary Table 22. Primers for quantitative PCR tests. *C. pomonella β-tubulin* or *actin* gene was used as reference to calculate relative expression levels of either *P450* or *Ors*. A 2^(-*ΔΔ*Ct) method was used for calculations.

Gene name	Primer	Sequence（5'-3'）	Product length (bp)
CYP6B2	F	TGAAGCGTGTATTAGATGAAGTG	188
	R	CAGCAGCAGACCTGATGG	
CYP6B2	F	ACTCGGGGGGGAGAGAACTGAAGGTC	
	R	TTCCTCGTCGGATATATCAGCCACG	
CpomOR3a	F	TGCTCTACATTGGACACCGAAG	156
	R	CCATACACTCCCAGGGCAAAT	
CpomOR3b	F	GTAAGTTTTATGGGCTGGTTTTT	142
	R	GCAGGTTTAGGGAAATTGTATAT	
β-tubulin	F	GCGGGAACCAGATTGGAGCTAA	267
	R	ACTGGCCGAACACGAAGTTGTC	
Actin	F	TCCACCAAAAAGCACCTACGGC	
	R	GGCGTGACCGAGGAGGAAGGT	

Supplementary Table 23. Genome resequencing three strains (Raz, Rv and S) of *Cydia pomonella* that have been resistance or susceptible to insecticides

Stains	Total read bases (bp)	Total reads
S	26,782,397,974	177,366,874
	25,950,921,608	171,860,408
	26,722,116,660	176,967,660
	29,235,471,494	193,612,394
	29,842,850,270	197,634,770
	29,120,591,298	192,851,598
Raz	25,759,973,652	170,595,852
	25,473,735,032	168,700,232
	24,600,420,794	162,916,694
	24,214,643,276	160,361,876
	25,513,180,762	168,961,462
	27,492,399,068	182,068,868
Rv	26,569,666,154	175,958,054
	29,283,719,920	193,931,920
	23,948,201,360	158,597,360
	23,374,581,956	154,798,556
	26,098,915,500	172,840,500
	24,591,382,538	162,856,838

Supplementary Table 24. Summary of the SNPs between resistant (Raz, resistance to azinphos methyl; and Rv, resistance to deltamethrin) and susceptible strains (S) of *Cydia pomonella*

Category	Raz	Rv
Total SNPs	9310882	8327401
Significant SNPs (p < 0.0001)	63549	155009
Intergenic SNPs	51884	126003
Protein-coding SNPs	11665	29006
Exons	1477	3908
Non-Synonymous	454	1245
Synonymous	1023	2663
In candidate loci	109	242

Supplementary Table 25. PCR confirmation of several SNPs significantly different between resistant and susceptible strains of *Cydia pomonella*. Nucleotide variation are tested using the designed primers on S, Rv and Raz strains. Ten individuals from each of the three strains were used for analysis and the PCR results of all samples are presented in the Fig. 6c.

Gene annotated	Forward primer (5'-3')	Reverse primer (5'-3')	Gene ID	Chromosome	Site	Nucleotide variation	Amino acid variation
Acetylcholinesterase (ACE1)	GGCCCATGATTGAATGTCTG	TTTGACCCTAAGAGAAGATTGG	CPOM02212	Z	24960009	T1114G	F372V
Voltage gated sodium channel (VGSC)	TAGAGAGCATGTGGGATTGC	AATTTCGTAGCCCTTGATCG	CPOM03720	17	15595715	C3244T	L1082F
Octopamine receptor (OAR1)	GTGAACCATGAAACTGGACCTAC	TAGGAAGGAGACCAATGCTG	CPOM08177	21	7548673	G207C	V73L
Octopamine receptor (OAR2)	GCTGGAGTGTGGATGTTGTC	CCTTCGCTCCTTTGATAGTG	CPOM18505	16	14997841	A897C	K299N
	GCTGGAGTGTGGATGTTGTC	CCTTCGCTCCTTTGATAGTG	CPOM18505	16	14997817	A873C	E291D
Aquaporin (AQP)	CCTACACCTCTGACCCGAGAA	CAGAATAATGCGTCTTCTTTGAC	CPOM20975	11	3322554	T277A	L93M
Muscarinic acetylcholine rece ptor (mAChR)	CTGAGTGGAACATGATCTGG	GATACAAGTCGCACGCTCTC	CPOM21680	Scaffold1163	4418	G208A	G70R
Cytochrome P450 (CYP6B2)	AGTCTCATTTTGTATGGG	ACATTAGCATGCAAATTC	CPOM05212	20	5259067	A(-52)T	--
	AGTCTCATTTTGTATGGG	ACATTAGCATGCAAATTC	CPOM05212	20	5259072	T(-57)C	--
	AGTCTCATTTTGTATGGG	ACATTAGCATGCAAATTC	CPOM05212	20	5259125	T(-110)G	--

Supplementary Table 26. SNPs in the genes potentially involved in chemical insecticide resistance analyzed via comparing to resistant and susceptible strains of *Cydia pomonella*

Genes /Gene families	Raz-S		Rv-S	
	Total (gene / exon)	Significant (gene / exon)	Total (gene / exon)	Significant (gene / exon)
P450	13730 / 2227	88 / 15	13360 / 2263	195 / 40
CCE	7877 / 1819	16 / 4	7053 / 1742	138 / 35
GST	3168 / 298	0 / 0	2689 / 281	34 / 1
ABC	12140 / 1309	22 / 4	11200 / 1282	229 / 28
nAChRs	1526 / 84	0 / 0	1443 / 78	0 / 0
mAChRs	252 / 41	0 / 0	328 / 57	22 / 1
ACE	464 / 17	6 / 1	381 / 11	15 / 2
AQP	933 / 93	2 / 1	832 / 86	8 / 2
GluCls	376 / 6	0 / 0	361 / 9	0 / 0
GABA	709 / 38	0 / 0	659 / 34	0 / 0
VGSC	432 / 23	0 / 0	170 / 41	3 / 1
VGCC	2101 / 143	0 / 0	1832 / 135	46 / 5
UGT	4146 / 722	23 / 4	3906 / 726	23 / 2
ICP	12237 / 1731	42 / 4	10983 / 1651	282 / 61
TRR	492 / 73	0 / 0	493 / 69	60 / 1
OAR	552 / 87	7 / 1	473 / 62	23 / 5
DAR	1209 / 117	0 / 0	928 / 99	3 / 0
TAR	271 / 18	0 / 0	253 / 20	0 / 0
POX	1596 / 135	0 / 0	1411 / 128	24 / 2
Other detox	16430 / 4225	287 / 73	15166 / 4173	209 / 61

CCE, carboxyesterase; GST, glutathione S-transferase; P450, cytochrome P450; ABC, ATP-binding cassette transporters; AQP, aquaporin; ACE, acetylcholinesterase; VGSC, voltage gated sodium channel; GABA, γ-Aminobutyrate gated chloride channel; GluCl, glutamate-gated chloride channel; VGCC, voltage gated calcium channel; mAChR, muscarinic acetylcholine receptor; nAChR, nicotinic acetylcholine receptor; OAR, octopamine receptor; TAR, tyramine receptor; DAR, dopamine receptor; TRR, tryptamine receptor; UGT, UDP-glucuronosyltransferase; ICP, insect cuticle proteins; POX, peroxidases; Other detox includes alcohol dehydrogenase, sulfotransferase, aldehyde oxidase, oxidoreductase, and fucosyltransferase

Supplementary Table 27. Transcriptomes used for OR gene expression analysis

Samples	NCBI SRA accession numbers
adult male antennae	SRX1082029
adult female antennae	SRX1082030
neonate larval heads	SRX1082032
adult	SRX371333
larval midgut	SRX532407
testis 1	SRX2068934
testis2	SRX2068933
accessory gland 1	SRX2068935
accessory gland 2	SRX2068936
male head	SRX2068938
ovary 1	SRX2068943
ovary 2	SRX2068944
female head	SRX2068932
female midgut 1	SRX2068939
female midgut 2	SRX2068940
male midgut 1	SRX2068941
male midgut 2	SRX2068942
Abdomen of female adult, abdomen of male adult, female pupa, female adult with hot treatment, female adult, male larva, one-day egg, four-day egg, five-instar larva and egg mix*	SRR8479433-SRR8479442

* These transcriptomes were from our own RNA-Seq data

Supplementary References

1. Coates, B. S., Abel, C. A. & Perera, O. P. Estimation of long terminal repeat element content in the *Helicoverpa zea* genome from high-throughput sequencing of bacterial artificial chromosome pools. *Genome* **60**, 310-324 (2017).
2. Li, R. et al. The sequence and *de novo* assembly of the giant panda genome. *Nature* **463**, 311-317 (2010).
3. Marcais, G. & Kingsford, C. A fast, lock-free approach for efficient parallel counting of occurrences of k-mers. *Bioinformatics* **27**, 764-770 (2011).
4. Chin, C. S. et al. Phased diploid genome assembly with single-molecule real-time sequencing. *Nat. Methods* **13**, 1050-1054 (2016).
5. Pryszcz, L. P. & Gabaldon, T. Redundans: an assembly pipeline for highly heterozygous genomes. *Nucleic Acids Res.* **44**, e113 (2016).
6. Walker, B. J. et al. Pilon: an integrated tool for comprehensive microbial variant detection and genome assembly improvement. *PLoS ONE* **9**, e112963 (2014).
7. Langmead, B. & Salzberg, S. L. Fast gapped-read alignment with Bowtie 2. *Nat. Methods* **9**, 357-359 (2012).
8. Servant, N. et al. HiC-Pro: an optimized and flexible pipeline for Hi-C data processing. *Genome Biol.* **16**, 259-269 (2015).
9. Burton, J. N. et al. Chromosome-scale scaffolding of *de novo* genome assemblies based on chromatin interactions. *Nat. Biotechnol.* **31**, 1119-1125 (2013).
10. Parra, G., Bradnam, K. & Korf, I. CEGMA: a pipeline to accurately annotate core genes in eukaryotic genomes. *Bioinformatics* **23**, 1061-1067 (2007).
11. Simao, F. A., Waterhouse, R. M., Ioannidis, P., Kriventseva, E. V. & Zdobnov, E. M. BUSCO: assessing genome assembly and annotation completeness with single-copy orthologs. *Bioinformatics* **31**, 3210-3212 (2015).
12. Yandell, M. & Ence, D. A beginner's guide to eukaryotic genome annotation. *Nat. Rev. Genet.* **13**, 329-342 (2012).
13. Benson, G. Tandem repeats finder: a program to analyze DNA sequences. *Nucleic Acids Res.* **27**, 573-580 (1999).
14. Smit, A. & Hubley, R. *RepeatModeler-1.0. 5.* **http://www.repeatmasker.org/RepeatModeler/** (2012).
15. Bao, Z. & Eddy, S. R. Automated *de novo* identification of repeat sequence families in sequenced genomes. *Genome Res.* **12**, 1269-1276 (2002).
16. Price, A. L., Jones, N. C. & Pevzner, P. A. *De novo* identification of repeat families in large genomes. *Bioinformatics* **21 Suppl 1**, i351-i358 (2005).
17. Tempel, S. Using and understanding RepeatMasker. *Methods Mol. Biol.* **859**, 29-51 (2012).
18. Liu, J., Xiao, H., Huang, S. & Li, F. OMIGA: Optimized Maker-Based Insect Genome Annotation. *Mol. Genet. Genomics* **289**, 567-573 (2014).

19. Trapnell, C. et al. Differential gene and transcript expression analysis of RNA-seq experiments with TopHat and Cufflinks. *Nat. Protoc.* **7**, 562-578 (2012).
20. Stanke, M., Steinkamp, R., Waack, S. & Morgenstern, B. AUGUSTUS: a web server for gene finding in eukaryotes. *Nucleic Acids Res.* **32**, W309-W312 (2004).
21. Korf, I. Gene finding in novel genomes. *BMC Bioinformatics* **5**, 59-67 (2004).
22. Lomsadze, A., Burns, P. D. & Borodovsky, M. Integration of mapped RNA-Seq reads into automatic training of eukaryotic gene finding algorithm. *Nucleic Acids Res.* **42**, e119 (2014).
23. Campbell, M. S., Holt, C., Moore, B. & Yandell, M. Genome annotation and curation using MAKER and MAKER-P. *Curr. Protoc. Bioinformatics* **48**, 4.11.11–14.11.39 (2014).
24. Jones, P. et al. InterProScan 5: genome-scale protein function classification. *Bioinformatics* **30**, 1236-1240 (2014).
25. Conesa, A. & Gotz, S. Blast2GO: A comprehensive suite for functional analysis in plant genomics. *Int. J. Plant Genomics* **2008**, 1-12 (2008).
26. Lowe, T. M. & Eddy, S. R. tRNAscan-SE: a program for improved detection of transfer RNA genes in genomic sequence. *Nucleic Acids Res.* **25**, 955-964 (1997).
27. Nawrocki, E. P., Kolbe, D. L. & Eddy, S. R. Infernal 1.0: inference of RNA alignments. *Bioinformatics* **25**, 1335-1337 (2009).
28. Guerra-Assuncao, J. A. & Enright, A. J. MapMi: automated mapping of microRNA loci. *BMC Bioinformatics* **11**, 133 (2010).
29. Griffiths-Jones, S., Grocock, R. J., van Dongen, S., Bateman, A. & Enright, A. J. miRBase: microRNA sequences, targets and gene nomenclature. *Nucleic Acids Res.* **34**, D140-D144 (2006).
30. Li, L., Stoeckert, C. J., Jr. & Roos, D. S. OrthoMCL: identification of ortholog groups for eukaryotic genomes. *Genome Res.* **13**, 2178-2189 (2003).
31. Rota-Stabelli, O., Daley, A. C. & Pisani, D. Molecular timetrees reveal a Cambrian colonization of land and a new scenario for ecdysozoan evolution. *Curr. Biol.* **23**, 392-398 (2013).
32. Misof, B. et al. Phylogenomics resolves the timing and pattern of insect evolution. *Science* **346**, 763-767 (2014).
33. Grabherr, M. G. et al. Genome-wide synteny through highly sensitive sequence alignment: *Satsuma*. *Bioinformatics* **26**, 1145-1151 (2010).
34. Bao, W., Kojima, K. K. & Kohany, O. Repbase update, a database of repetitive elements in eukaryotic genomes. *Mob. DNA* **6**, 11-16 (2015).
35. Tarailo-Graovac, M. & Chen, N. Using RepeatMasker to identify repetitive elements in genomic sequences. *Curr. Protoc. Bioinformatics* **25**, 4.10.11-14.10.14 (2009).
36. Novak, P., Neumann, P. & Macas, J. Graph-based clustering and characterization of repetitive sequences in next-generation sequencing data. *BMC Bioinformatics* **11**, 378 (2010).

37. Novak, P., Neumann, P., Pech, J., Steinhaisl, J. & Macas, J. RepeatExplorer: a Galaxy-based web server for genome-wide characterization of eukaryotic repetitive elements from next-generation sequence reads. *Bioinformatics* **29**, 792-793 (2013).
38. Fukova, I., Nguyen, P. & Marec, F. Codling moth cytogenetics: karyotype, chromosomal location of rDNA, and molecular differentiation of sex chromosomes. *Genome* **48**, 1083-1092 (2005).
39. Nguyen, P. et al. Neo-sex chromosomes and adaptive potential in tortricid pests. *Proc. Natl. Acad. Sci. U. S. A.* **110**, 6931-6936 (2013).
40. Kurtz, S. et al. Versatile and open software for comparing large genomes. *Genome Biol.* **5**, R12 (2004).
41. Krieger, J., Grosse-Wilde, E., Gohl, T. & Breer, H. Candidate pheromone receptors of the silkmoth *Bombyx mori*. *Eur. J. Neurosci.* **21**, 2167-2176 (2005).
42. Nakagawa, T., Sakurai, T., Nishioka, T. & Touhara, K. Insect sex-pheromone signals mediated by specific combinations of olfactory receptors. *Science* **307**, 1638-1642 (2005).
43. Wanner, K. W. et al. Female-biased expression of odourant receptor genes in the adult antennae of the silkworm, *Bombyx mori*. *Insect Mol. Biol.* **16**, 107-119 (2007).
44. Tanaka, K. et al. Highly selective tuning of a silkworm olfactory receptor to a key mulberry leaf volatile. *Curr. Biol.* **19**, 881-890 (2009).
45. Engsontia, P., Sangket, U., Chotigeat, W. & Satasook, C. Molecular evolution of the odorant and gustatory receptor genes in lepidopteran insects: implications for their adaptation and speciation. *J. Mol. Evol.* **79**, 21-39 (2014).
46. Koenig, C. et al. A reference gene set for chemosensory receptor genes of *Manduca sexta*. *Insect Biochem. Mol. Biol.* **66**, 51-63 (2015).
47. Zhan, S., Merlin, C., Boore, J. L. & Reppert, S. M. The monarch butterfly genome yields insights into long-distance migration. *Cell* **147**, 1171-1185 (2011).
48. Eddy, S. R. Accelerated profile HMM searches. *PLoS Comput. Biol.* **7**, e1002195 (2011).
49. Finn, R. D. et al. The Pfam protein families database: towards a more sustainable future. *Nucleic Acids Res.* **44**, D279-D285 (2016).
50. Krogh, A., Larsson, B., von Heijne, G. & Sonnhammer, E. L. Predicting transmembrane protein topology with a hidden Markov model: application to complete genomes. *J. Mol. Biol.* **305**, 567-580 (2001).
51. Zhou, X. et al. Phylogenetic and transcriptomic analysis of chemosensory receptors in a pair of divergent ant species reveals sex-specific signatures of odor coding. *PLoS Genet.* **8**, e1002930 (2012).
52. Katoh, K. & Standley, D. M. MAFFT multiple sequence alignment software version 7: improvements in performance and usability. *Mol. Biol. Evol.* **30**, 772-780 (2013).
53. Capella-Gutierrez, S., Silla-Martinez, J. M. & Gabaldon, T. trimAl: a tool for automated alignment trimming in large-scale phylogenetic analyses. *Bioinformatics* **25**, 1972-

1973 (2009).
54. Stamatakis, A. RAxML version 8: a tool for phylogenetic analysis and post-analysis of large phylogenies. *Bioinformatics* **30**, 1312-1313 (2014).
55. Li, B. & Dewey, C. N. RSEM: accurate transcript quantification from RNA-Seq data with or without a reference genome. *BMC Bioinformatics* **12**, 323 (2011).
56. Yang, K., Huang, L. Q., Ning, C. & Wang, C. Z. Two single-point mutations shift the ligand selectivity of a pheromone receptor between two closely related moth species. *Elife* **6**, e29100 (2017).
57. Wang, G., Carey, A. F., Carlson, J. R. & Zwiebel, L. J. Molecular basis of odor coding in the malaria vector mosquito *Anopheles gambiae*. *Proc. Natl. Acad. Sci. U. S. A.* **107**, 4418-4423 (2010).
58. Sauphanor, B., Bovier, J. C. & Brosse, V. Spectrum of insecticide resistance in *Cydia pomonella* (Lepidoptera : Tortricidae) in southeastern France. *J. Econ. Entomol.* **91**, 1225-1231 (1998).
59. Guennelon, G., Audemard, H., Fremond, J. C. & Ammari, M. A. E. Permanent rearing of codling moth on an artificial medium. *Agronomie* **1**, 59-64 (1981).
60. Sauphanor, B. et al. Monitoring resistance to diflubenzuron and deltamethrin in French codling moth populations (*Cydia pomonella*). *Pest Manag. Sci.* **56**, 74-82 (2000).
61. Andrews, S. *FastQC: a quality control tool for high throughput sequence data*. **https://www.bioinformatics.babraham.ac.uk/projects/fastqc/** (2010).
62. Bolger, A. M., Lohse, M. & Usadel, B. Trimmomatic: a flexible trimmer for Illumina sequence data. *Bioinformatics* **30**, 2114-2120 (2014).
63. Li, H. & Durbin, R. Fast and accurate short read alignment with Burrows-Wheeler transform. *Bioinformatics* **25**, 1754-1760 (2009).
64. Li, H. et al. The sequence alignment/map format and SAMtools. *Bioinformatics* **25**, 2078-2079 (2009).
65. Danecek, P. & McCarthy, S. A. BCFtools/csq: haplotype-aware variant consequences. *Bioinformatics* **33**, 2037-2039 (2017).
66. Faucon, F. et al. Unravelling genomic changes associated with insecticide resistance in the dengue mosquito *Aedes aegypti* by deep targeted sequencing. *Genome Res.* **25**, 1-13 (2015).
67. Turner, S. D. qqman: an R package for visualizing GWAS results using QQ and manhattan plots. Preprint at **https://doi.org/10.1101/005165** (2014).
68. Tang, R. et al. Identification and testing of oviposition attractant chemical compounds for *Musca domestica*. *Sci. Rep.* **6**, 33017 (2016).
69. Wu, H. et al. Specific olfactory neurons and glomeruli are associated to differences in behavioral responses to pheromone components between two *Helicoverpa* species. *Front. Behav. Neurosci.* **9**, 206 (2015).
70. Tang, R., Su, M. W. & Zhang, Z. N. Electroantennogram responses of an invasive species fall webworm (*Hyphantria cunea*) to host volatile compounds. *Chin. Sci. Bull.*

57, 4560-4568 (2012).
71. Chang, H. et al. A pheromone antagonist regulates optimal mating time in the moth *Helicoverpa armigera*. *Curr. Biol.* **27**, 1610-1615 (2017).
72. Tadi'C, M. D. *The Biology of the Codling Moth (Carpocapsa pomomella L.) as the Basis for Its Control* (University of Belgrade Press, Belgrade, 1957).
73. Slingerland, M. V. *Codling Moth* (Cornell University Press, New York, 1898).
74. Simpson, C. B. The codling moth. *USDA, Division of Entomology*, 1-105 (1903).
75. Simpson, C. B. Report on codling moth investigations in the northwest during 1901. (1902).
76. Lounsbury, C. P. Codling moth. *Agricultural Journal* **13**, 597-616 (1898).
77. Cotton, A. D. Report on the occurrence of insect and fungus pests on plants in England and Wales in the year 1917. In: *Miscellaneous Publications. Ministry of Agriculture and Fisheries* (1918).
78. Evans, H. H. Codling moth control in British Columbia. *Agric. J.* **6**, 170-171 (1921).
79. Woodworth, C. W. Codling moth control in the Sacramento Valley. *Univy. of California, Coll. of Agric. Circ.*, 4 (1913).
80. Sanderson, E. D. *Insect Pests of Farm, Garden and Orchard* (John Wiley and Sons Press, New York, 1912).
81. Hall, J. A. Six years' study of the life history and habits of the codling moth (*Carpocapsa pomonella* L.). *59th Annual Report Entomological Society of Ontario 1928.* **59**, 96-105 (1929).
82. Fernald, H. T. & Bourne, A. I. *32nd Annual report mass* (1919).
83. Lehmann, H. The outbreak of *Aporia crataegi* and the organisation for combating it. *Flugschr. Deutsch. Ges. angew. Ent.*, 31 (1922).
84. Jack, R. W. Insect pests of fruits other than citrus in southern Rhodesia. *Rhodesia Agricultural Journal* **19**, 569-582 (1922).
85. Author. Fruit fly surveys in Spain, Portugal and Italy. In: *Service and Regulatory Announcements.* 18-20 (Federal Horticultural Board, 1925).
86. Wilkinson, D. S. *Cyprus: Ann. Rept. Dir. Agric. 1925* (1926).
87. Macdougall, R. S. The apple fruit moth or "miner" (*Argyresthia conjugella*, Zeller). *Scottish Journal of Agriculture* **9**, 7 (1926).
88. Cutright, C. R. Codling moth biology and control investigations. *Ohio Agricultural Experiment Station* (1937).
89. Xu, J., Liu, W., Liu, H., Wu, L. F. & Zhang, R. Z. Spread and impact of the codling moth *Cydia pomonella* (L.) in China. *Journal of Biosafety* **24**, 327-336 (2015).
90. Khajuria, D. R., Sharma, J. P. & Dogra, G. S. Incidence of apple fruit moth (*Argyresthia conjugella* Zell.) in Himachal Pradesh, India. *Trop. Pest Manag.* **32**, 350-350 (1986).
91. Allman, S. L. The codling moth (*Cydia pornonella* L.). *Sci. Bull. Dep. Agric. N. S.W.* **31**, 36 (1928).
92. Flint, W. P. The oriental fruit moth, curculio and codling moth in Illinois in 1929. *Trans.*

Illinois Hort. Soc. **63**, 139-146 (1930).
93. Anonymous. Studies of insect pests and related matters. In: *43rd Annual Report South Carolina Experimental Station*, 59-74 (1930).
94. Vappula, N. A. The occurrence of injurious animals in Finland in 1933. *Valtion Maatalouskoetoiminnan Tiedonantoja* **24**, 349 (1935).
95. Askew, R. R. On the biology and taxonomy of some European species of the genus *Elachertus* Spinola (Hymenoptera, Eulophidae). *Bull. Entomol. Res.* **55**, 53 (1964).
96. Iraqui, S. E. & Hmimina, M. H. Assessment of control strategies against *Cydia pomonella* (L.) in Morocco. *J. Plant Prot. Res.* **56**, 82-88 (2016).
97. Staehelin, M. & Bovey, P. Control of codling moth and scab in apples and pears in French Switzerland. *Annu. agric. Suisse*, 635-680 (1940).
98. Steiner, L. F. *Transactions of the Indiana horticultural society* (Holloway, Douglas, 1920).
99. Das, S. A case of commensalism between a lamellibranch and a monascidian. *Curr. Sci.* **7**, 114-115 (1938).
100. Janjua, N. A. Codling moth in Afghanistan. *Curr. Sci.* **7**, 115-116 (1938).
101. Lekic, M. B. The biology of the codling moth on the territory of the Serbian People's Republic and measures for its control. *Plant Protection* **1**, 32-65 (1950).
102. Paternotte, E. Apple and pear grub *Cydia pomonella* L. *Fruitteelt (Den Haag)* **2**, 7-8 (1989).
103. Zhang, X. Z. The new discovery of *Carpocapsa pomonella* L. in China. *Acta Entomol. Sin.* **7**, 467-472 (1957).
104. Wang, L. et al. Dynamic state of *Laspeyresia pomonella* and release techniques of *Trichogramma* in orchards. *Xinjiang Agricultural Sciences* **48**, 261-265 (2011).
105. Zhu, H., Kumar, S. & Neven, L. G. Codling moth (Lepidoptera: Tortricidae) establishment in China: stages of invasion and potential future distribution. *J. Insect Sci.* **17**, 85 (2017).
106. Mansour, M. Attract and kill for codling moth *Cydia pomonella* (Linnaeus) (Lepidoptera: Tortricidae) control in Syria. *J. Appl. Entomol.* **134**, 234-242 (2010).
107. Geier, P. W. The life history of codling moth, *Cydia Pomonella* (L.) (Lepidoptera:Tortricidae), in the Australian capital territory. *Aust. J. Zool.* **11**, 323-367 (1963).
108. Wearing, C. H., Hansen, J. D., Whyte, C., Miller, C. E. & Brown, J. The potential for spread of codling moth (Lepidoptera: Tortricidae) via commercial sweet cherry fruit: a critical review and risk assessment. *Crop Prot.* **20**, 465-488 (2001).
109. Vavrovič, J., Svobodová, E., Eliašová, M., Kollár, J. & Šiška, B. Model estimation of potential infestation pressure of codling moth (*Cydia pomonella*) in condition of changing climate in Slovakia. *Mendel and bioclimatology*, 3-5 (2014).
110. Bodor, J. Separation of the moth pests of peach on the basis of imago and larvae. *Novenyvedelmi kutato Intezet Kozlemenyei* **3**, 3961 (1969).

111. Rosenberg, H. T. The biology and distribution in France of the larval parasites of *Cydia pomonella*, L. *Bull. Entomol. Res.* **25**, 201 (1934).
112. El-Gamil, F. M., Gaaboub, I. A. & El-Sawaf, S. K. Population study of codling moth *Cydia* (*Carpocaps = Laspeyresia*) *Pomonella* L. (Olethreutidae, Lepidoptera) infesting pear orchards in the eastern outskirts of Alexandria, Egypt. *J. Agric. Sci.* **89**, 655-657 (1977).
113. Komarek, S. Study on the species composition and the population dynamics of the family Tortricidae in some orchards in southern Bohemia using pheromone traps. *Pflanzenschutzberichte* **48**, 2-23 (1987).
114. Kozlowski, J. Forecasting the occurrence and establishing the control date of the codling moth (*Carpocapsa pomonella*) in Wielkopolska in 1981-92. *Prace Naukowe Instytutu Ochrony Roslin* **35**, 43-47 (1994).
115. Subinprasert, S. Natural enemies and their impact on overwintering codling moth populations (*Laspeyresia pomonella* L.) (Lep., Tortricidae) in South Sweden. *J. Appl. Entomol.* **103**, 46-55 (2010).
116. Zhigarevich, G. P. & Yakubov, Z. B. Phenology of the codling moth and times for its control. *Sadovodstvo I Vinogradarstvo*, 18-20 (1990).
117. Ahmad, T. R. Degree-days requirements for predicting emergence and flight of the codling moth *Cydia pomonella* (L) (Lep, Olethreutidae). *Journal of Applied Entomology-Zeitschrift Fur Angewandte Entomologie* **106**, 345-349 (1988).
118. Minoiu, N. & Boaru, M. Utilisation of some synthetic insect pheromones in orchards of north-eastern Transylvania. *Probleme De Protecţia Plantelor* **38**, 337-346 (1989).
119. Steinberg, S., Podoler, H. & Applebaum, S. Studies on the diapause of the codling moth in Israel; I. Diapause induction in field populations in Upper Galilee. *Hassadeh* **68**, 682-686 (1988).
120. Pawar, A. D. & Tuhan, N. C. Codling moth (Lepidoptera: Olethreutidae) - suppression by male removal with sex pheromone traps in Ladakh, Jammu and Kashmir. *Indian J. Entomol.* **47**, 226-229 (1985).
121. Stefanovska, T. R., Shelestova, V. S., Pidlisnyuk, V. K. & Goncharenko, O. I. *The Use of Biological Control for Cydia pomonella L. (Lepidoptera: Tortricidae) Management in Ukraine* (University of California Press, Berkeley, 2000).
122. Minks, A. K. Mating disruption of the codling moth. In: *Insect Pheromone Research* (eds Cardé, R. T., Minks, A. K.) 372-376 (Springer Press, Boston, 1997).
123. Zhao, L. et al. Mapping the disjunct distribution of introduced codling moth *Cydia pomonella* in China. *Agricultural & Forest Entomology* **17**, 214-222 (2015).
124. Qin, X. H., Ma, D. C., Zhang, Y., Li, G. H. & Wang, P. Harm of *Laspeyresia pomonella* (L.) in northwest of China. *Plant Quarantine* **20**, 2 (2006).
125. Kovaleski, A. & Mumford, J. Pulling out theevil by the root: the codling moth *Cydia pomonella* eradication programme in Brazil. In: *Area-Wide Control of Insect Pests* (eds Vreysen, M. J. B., Robinson, A. S., Hendrichs, J.) 591-601 (Springer Press, Dordrecht,

2007).
126. Giner, M., Balcells, M. & Avilla, J. Antenna elicitation and behavioral responses of oriental fruit moth, *Grapholita molesta*, to allyl cinnamate. *Afr. J. Biotechnol.* **13**, 4536-4540 (2014).
127. Asmatullah-Kakar & Hazara, A. H. Non-chemical treatments for control of codling moth *Cydia pomonella* L. (Lepidoptera: Tortricidae) in Quetta Valley, Balochistan, Pakistan. *Pak. J. Zool.* **41**, 189-196 (2009).
128. Polesny, F. Integrated control of codling moth (*Cydia Pomonella*) in Austria. *Acta Horticulturae*, 285-290 (2000).
129. Liu, Y. Y., Luo, J. C., Zhou, Z. X. & Wei, Y. Y. Life tables of the experimental population of codling moth, *Cydia pomonella* (L.) at different temperatures. *Journal of Plant Protection* **39**, 205-210 (2012).
130. Zhang, R. Z. et al. Progress on monitoring and control of the codling moth, *Cydia pomonella* (L.). *Chinese Journal of Applied Entomology* **49**, 37-42 (2012).
131. Willett, M. J., Neven, L. & Miller, C. E. The occurrence of codling moth in low latitude countries:validation of pest distribution reports. *HortTechnology* **19**, 633 (2009).
132. Wang, H. M. Occurrence characteristics and control countermeasures of codling moth in Zhongning county. *Inner Mongolia Agricultural Science And Technology*, 70-71 (2014).
133. Zichová, T., Falta, V., Kocourek, F. & Stará, J. Differences in the susceptibility of codling moth populations to *Cydia pomonella* granulovirus in the Czech Republic. *Horticultural Science* **38**, 21-26 (2011).
134. Cook, W. C. Studies on the flight of nocturnal Lepidoptera. In: *18th Report Minnesota State Entomologist, Agricultural Experimental Station* (eds Cook, W. C.) 43-56 (University Farm St. Paul Press, Minnesota, 1921).
135. Haseman, L. The codling moth problem in Missouri. *Bull. Mo. agric. Exp. Stn.* **334**, 16 (1934).
