# Supplementary material for: A chromosome-level genome assembly of Cydia pomonella provides insights into chemical ecology and insecticide resistance
Source: Nat Commun. 2019 Sep 17;10:4237. doi: 10.1038/s41467-019-12175-9 (PMC6748993; doi:10.1038/s41467-019-12175-9)
Supplement: Supplementary file 3 — Reporting Summary [file 41467_2019_12175_MOESM3_ESM.pdf]

nature research

Corresponding author(s): Fei Li

Last updated by author(s): Jul 29, 2019

Reporting Summary

Nature Research wishes to improve the reproducibility of the work that we publish. This form provides structure for consistency and transparency in reporting. For further information on Nature Research policies, see Authors & Referees and the Editorial Policy Checklist.

Statistics

For all statistical analyses, confirm that the following items are present in the figure legend, table legend, main text, or Methods section.

n/a	Confirmed	
☐	☒	The exact sample size (*n*) for each experimental group/condition, given as a discrete number and unit of measurement
☐	☒	A statement on whether measurements were taken from distinct samples or whether the same sample was measured repeatedly
☐	☒	The statistical test(s) used AND whether they are one- or two-sided<br>*Only common tests should be described solely by name; describe more complex techniques in the Methods section.*
☐	☒	A description of all covariates tested
☐	☒	A description of any assumptions or corrections, such as tests of normality and adjustment for multiple comparisons
☐	☒	A full description of the statistical parameters including central tendency (e.g. means) or other basic estimates (e.g. regression coefficient) AND variation (e.g. standard deviation) or associated estimates of uncertainty (e.g. confidence intervals)
☐	☒	For null hypothesis testing, the test statistic (e.g. *F*, *t*, *r*) with confidence intervals, effect sizes, degrees of freedom and *P* value noted<br>*Give P values as exact values whenever suitable.*
☐	☒	For Bayesian analysis, information on the choice of priors and Markov chain Monte Carlo settings
☐	☒	For hierarchical and complex designs, identification of the appropriate level for tests and full reporting of outcomes
☐	☒	Estimates of effect sizes (e.g. Cohen's *d*, Pearson's *r*), indicating how they were calculated

Our web collection on statistics for biologists contains articles on many of the points above.

Software and code

Policy information about availability of computer code

Data collection	*Provide a description of all commercial, open source and custom code used to collect the data in this study, specifying the version used OR state that no software was used.*
Data analysis	*Provide a description of all commercial, open source and custom code used to analyse the data in this study, specifying the version used OR state that no software was used.*

For manuscripts utilizing custom algorithms or software that are central to the research but not yet described in published literature, software must be made available to editors/reviewers. We strongly encourage code deposition in a community repository (e.g. GitHub). See the Nature Research guidelines for submitting code & software for further information.

Data

Policy information about availability of data

All manuscripts must include a data availability statement. This statement should provide the following information, where applicable:

- Accession codes, unique identifiers, or web links for publicly available datasets
- A list of figures that have associated raw data
- A description of any restrictions on data availability

The sequence data from the Cydia genome project have been deposited in the GenBank under the accession number GCA_003425675.2[https://www.ncbi.nlm.nih.gov/assembly/GCA_003425675.2]. The BioProject of the Cydia genome project is PRJNA464426[https://www.ncbi.nlm.nih.gov/bioproject/PRJNA464426] and WGS project is QFTL02[https://www.ncbi.nlm.nih.gov/nuccore/QFTL00000000.2]. The BioSample used for genome sequencing is SAMN09205828[https://www.ncbi.nlm.nih.gov/biosample/SAMN09205828]. The genome resequencing data of resistant strains have been deposited in the GenBank under SRR8479443-SRR8479460 and the transcriptome data have been deposited in SRA under SRR8479433-SRR8479442. The source data underlying Figs 3c, 4a–d, 6d-f, and Supplementary Fig 14 are provided as a Source Data file. All data mentioned in this paper can also be accessed at www.insect-genome.com/cydia/. All other relevant data is available upon request.

Field-specific reporting

Please select the one below that is the best fit for your research. If you are not sure, read the appropriate sections before making your selection.

☒ Life sciences ☐ Behavioural & social sciences ☐ Ecological, evolutionary & environmental sciences

For a reference copy of the document with all sections, see nature.com/documents/nr-reporting-summary-flat.pdf

Life sciences study design

All studies must disclose on these points even when the disclosure is negative.

Sample size	For genome re-sequencing, six individual were used for each strain. For RNA interference experiments, 30 individuals were used for study in each replicate. For each EAG experiments, we tested 16 individuals and repeated for 10 times. We did Pearson analysis to ensure the sample size is enough for our analysis.
Data exclusions	No data exclusion in our analyses.
Replication	For gene expression analysis and RNA interference, all experiments were repeated for three-five replicates. For EAG experiment, we repeated for 10 times.
Randomization	Randomization sample allocation were used for gene expression and RNA interference experiments by random assigning the selected individuals to different groups.
Blinding	Blind experiments are not widely used in gene function studies of insects. In our experiments, we random named the groups (negative control or treated groups), and the investigators who collected the RNAi results did not know which group was RNAi-treated.

Reporting for specific materials, systems and methods

We require information from authors about some types of materials, experimental systems and methods used in many studies. Here, indicate whether each material, system or method listed is relevant to your study. If you are not sure if a list item applies to your research, read the appropriate section before selecting a response.

Materials & experimental systems

n/a	Involved in the study	
☒	☐	Antibodies
☒	☐	Eukaryotic cell lines
☒	☐	Palaeontology
☐	☒	Animals and other organisms
☒	☐	Human research participants
☒	☐	Clinical data

Methods

n/a	Involved in the study	
☒	☐	ChIP-seq
☒	☐	Flow cytometry
☒	☐	MRI-based neuroimaging

Animals and other organisms

Policy information about studies involving animals; ARRIVE guidelines recommended for reporting animal research

Laboratory animals	codling moth
Wild animals	we did not use wild animals
Field-collected samples	we did not use field-collected samples.
Ethics oversight	The studies in this work have received ethical approval from the board of ethic committee, institute of plant protection, Chinese Academy of Agriculture Science, Beijing, China

Note that full information on the approval of the study protocol must also be provided in the manuscript.
