# Supplementary material for: A chromosome-level genome assembly of Cydia pomonella provides insights into chemical ecology and insecticide resistance
Source: Nat Commun. 2019 Sep 17;10:4237. doi: 10.1038/s41467-019-12175-9 (PMC6748993; doi:10.1038/s41467-019-12175-9)
Supplement: Supplementary file 4 — Description of Additional Supplementary Files [file 41467_2019_12175_MOESM4_ESM.pdf]

Description of Additional Supplementary Files

File Name: Supplementary Data 1
Description: The chromosome positions of 667 genes that putatively associated with chemical insecticide resistance.
